# HSP70-mediated mitochondrial dynamics and autophagy represent a novel vulnerability in pancreatic cancer

**DOI:** 10.1038/s41418-024-01310-9

**Published:** 2024-05-28

**Authors:** Giulia D. S. Ferretti, Colleen E. Quaas, Irene Bertolini, Alessandro Zuccotti, Ozge Saatci, Jennifer A. Kashatus, Salma Sharmin, David Y. Lu, Adi Narayana Reddy Poli, Abigail F. Quesnelle, Jezabel Rodriguez-Blanco, Aguirre A. de Cubas, G. Aaron Hobbs, Qin Liu, John P. O’Bryan, Joseph M. Salvino, David F. Kashatus, Ozgur Sahin, Thibaut Barnoud

**Affiliations:** 1 Department of Biochemistry and Molecular Biology, Medical University of South Carolina, Charleston, SC USA; 2 Hollings Cancer Center, Medical University of South Carolina, Charleston, SC USA; 3 Molecular and Cellular Oncogenesis Program, The Wistar Institute, Philadelphia, PA USA; 4 Department of Microbiology, Immunology, and Cancer Biology, University of Virginia Health System, Charlottesville, VA USA; 5 Darby Children’s Research Institute, Department of Pediatrics, Medical University of South Carolina, Charleston, SC USA; 6 Department of Microbiology and Immunology, Medical University of South Carolina, Charleston, SC USA; 7 Department of Cell and Molecular Pharmacology and Experimental Therapeutics, Medical University of South Carolina, Charleston, SC USA; 8 Ralph H. Johnson VA Medical Center, Charleston, SC USA

**Keywords:** Molecular biology, Cancer models

## Abstract

Pancreatic ductal adenocarcinoma (PDAC), the most prevalent type of pancreatic cancer, is one of the deadliest forms of cancer with limited therapy options. Overexpression of the heat shock protein 70 (HSP70) is a hallmark of cancer that is strongly associated with aggressive disease and worse clinical outcomes. However, the underlying mechanisms by which HSP70 allows tumor cells to thrive under conditions of continuous stress have not been fully described. Here, we report that PDAC has the highest expression of HSP70 relative to normal tissue across all cancers analyzed. Furthermore, HSP70 expression is associated with tumor grade and is further enhanced in metastatic PDAC. We show that genetic or therapeutic ablation of HSP70 alters mitochondrial subcellular localization, impairs mitochondrial dynamics, and promotes mitochondrial swelling to induce apoptosis. Mechanistically, we find that targeting HSP70 suppresses the PTEN-induced kinase 1 (PINK1) mediated phosphorylation of dynamin-related protein 1 (DRP1). Treatment with the HSP70 inhibitor AP-4-139B was efficacious as a single agent in primary and metastatic mouse models of PDAC. In addition, we demonstrate that HSP70 inhibition promotes the AMP-activated protein kinase (AMPK) mediated phosphorylation of Beclin-1, a key regulator of autophagic flux. Accordingly, we find that the autophagy inhibitor hydroxychloroquine (HCQ) enhances the ability of AP-4-139B to mediate anti-tumor activity in vivo. Collectively, our results suggest that HSP70 is a multi-functional driver of tumorigenesis that orchestrates mitochondrial dynamics and autophagy. Moreover, these findings support the rationale for concurrent inhibition of HSP70 and autophagy as a novel therapeutic approach for HSP70-driven PDAC.

## Introduction

The majority of human cancers remain refractory to chemotherapies in large part because of their resistance to apoptosis. Several proteins are known to exert anti-apoptotic functions, including the heat shock protein 70 (HSP70) [1]. HSP70 (also known as HSPA1A) is a protein chaperone that is overexpressed in a wide range of human cancers and is associated with poor survival [2]. HSP70 plays a role in several pro-survival mechanisms, including protein folding, assembly of protein complexes, transport of proteins across membranes, and targeting damaged proteins for degradation [3]. By regulating protein quality control, HSP70 allows tumor cells to survive the harsh conditions they encounter within the tumor microenvironment (TME) [2, 4–7]. In addition, HSP70 provides tumors a selective advantage by interfering with anti-tumor immunity and promoting metastasis [8]. Thus, HSP70 is considered to act as an oncogene that is essential for the initiation and progression of human cancer [2]. Notably, HSP70 is highly expressed in tumor cells but is virtually undetectable in unstressed cells and tissues [2, 4–7]. As a result, the tumor-specific expression of HSP70 has rendered this chaperone an attractive target for cancer therapy.

Pancreatic ductal adenocarcinoma (PDAC), the most prevalent type of pancreatic cancer, is an aggressive and fatal malignancy. In 2022, over 465,000 deaths from pancreatic cancer were reported worldwide [9]. Approximately ninety percent of PDACs are associated with mutations in Kirsten rat sarcoma (*KRAS*) and as a result are considered to be the most “RAS addicted” of all human cancers. Recent advances have shifted the paradigm of the undruggable RAS protein. A ground-breaking inhibitor of KRAS, sotorasib, was approved in 2021 by the U.S. Food and Drug Administration for certain cancers harboring a KRAS G12C mutation [10]. However, KRAS G12C mutations account for only 1–2% of pancreatic cancers [11]. Alternative therapies have been extensively investigated in PDAC, including the inhibition of the extracellular signal-regulated kinase/mitogen-activated protein kinase (ERK-MAPK) pathway that is constitutively activated by mutant RAS. Unfortunately, pharmacological inhibition of the ERK-MAPK pathway has proven largely ineffective in patients with PDAC [12]. Furthermore, clinical trials using immune checkpoint blockade against PD-1 and/or CTLA-4 have also proven ineffective in PDAC [13–15]. These results highlight the urgent need for novel therapeutic strategies to combat the intrinsically resistant nature of PDAC.

We previously reported that a significant fraction of the cytosolic HSP70 localizes to the mitochondria of PDAC cells; in contrast, normal cells and tissues do not show mitochondrial localization of HSP70 [3]. However, little is known about the role of HSP70 at the mitochondria of tumor cells. Furthermore, whether mitochondrial HSP70 represents a novel vulnerability in PDAC has yet to be determined. In this study, we demonstrated that HSP70 inhibition altered mitochondrial subcellular localization and induced mitochondrial swelling to promote apoptosis in PDAC cells. Mechanistically, we found that targeting HSP70 inhibited the PINK1-mediated phosphorylation of DRP1 and suppressed mitochondrial dynamics, a process that is essential for the progression of PDAC [16]. Treatment with the HSP70 inhibitor AP-4-139B showed single-agent efficacy in multiple xenograft models, and in a model of metastasis by suppressing the epithelial-mesenchymal transition (EMT). We also found that HSP70 inhibition promoted the AMPK-mediated phosphorylation of Beclin-1, a critical regulator of autophagy. In line with these findings, HSP70 inhibition increased autophagic flux in PDAC cells and synergized with the autophagy inhibitor hydroxychloroquine (HCQ) in vivo. Together, our data suggest that HSP70 inhibition may serve as a novel therapeutic strategy in PDAC that may be further enhanced with concomitant inhibition of autophagy.

## Results

### HSP70 inhibition impairs mitochondrial function in PDAC cells

**Fig. 1:**
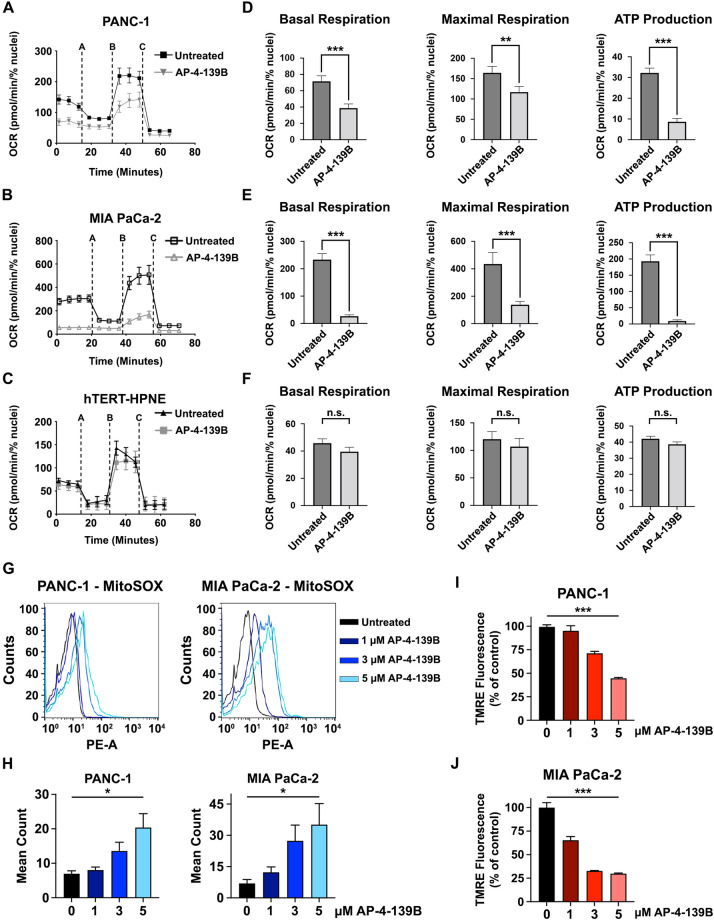
HSP70 inhibition impairs mitochondrial function and induces ROS. **A**–**C** Mitochondrial oxygen consumption rates (OCR) were measured in pancreatic cancer cell lines (PANC-1 and MIA PaCa-2), as well as non-transformed pancreatic ductal cells (hTERT-HPNE), in the presence or absence of 500 nM of HSP70i (AP-4-139B). All experiments were performed in triplicate, with each group containing 8–10 technical replicates. **D**–**F** Basal oxygen consumption rates (OCR), maximal respiration, and ATP production were analyzed and quantified in the presence or absence of 500 nM AP-4-139B. **p < 0.01, ***p < 0.001, n.s. not significant. **G**–**H** PANC-1 and MIA PaCa-2 cells were treated with the indicated doses of AP-4-139B for 48 h. Cells were then incubated with MitoSOX-Green, harvested, and analyzed by flow cytometry to determine mitochondrial ROS production. Fluorescence mean was analyzed and plotted on a histogram. *p < 0.05; n = 3 independent experiments. PANC-1 (**I**) and MIA PaCa-2 (**J**) cells were treated with the indicated doses of AP-4-139B for 24 h. Cells were then stained with 50 nM of TMRE for 30 min, and fluorescence intensity was measured using a plate reader. Shown are representative data of two independent experiments, with each condition containing six technical replicates. ***p < 0.001.

Unlike normal cells, tumor cells have a significant fraction of HSP70 localized to the mitochondria [3]. We sought to target this potential vulnerability in PDAC by leveraging a uniquely acting HSP70 inhibitor, AP-4-139B (also ‘139B’), that targets HSP70 in multiple compartments of tumor cells including the mitochondria [17]. AP-4-139B is a highly specific inhibitor that targets the stress-induced HSP70. Notably, AP-4-139B does not bind to other HSP70 family members that are constitutively expressed and not appreciably induced by stress, including GRP75 and BiP [17]. To test the impact of AP-4-139B on mitochondrial activity in PDAC, we assessed mitochondrial function in TERT-immortalized human pancreatic epithelial cells (hTERT-HPNE) versus two PDAC cell lines, PANC-1 and MIA PaCa-2. Each cell line was incubated for 24 h with a sub-lethal concentration of AP-4-139B (500 nM) and subsequently subjected to the Mitochondrial Stress Test using a Seahorse XFe96 analyzer. These analyses showed that non-transformed hTERT-HPNE cells were largely unaffected by HSP70 inhibition (HSP70i). However, PANC-1 and MIA PaCa-2 cells showed a significant reduction in basal and maximal oxygen consumption rates, along with a decrease in ATP production when treated with AP-4-139B (Fig. 1A–F).

HSP70 has been shown to suppress mitochondrial reactive oxygen species (ROS) [18]. Given that large changes in ROS can promote oxidative stress to induce cell death [19], we sought to determine whether HSP70 inhibition impacts the production of global intracellular ROS as well as mitochondrial ROS (mROS). To test this, we performed CellROX and MitoSOX-based flow cytometric assays to detect ROS and mROS in untreated versus AP-4-139B treated PDAC cells, respectively. We found that HSP70 inhibition increased ROS levels (Supplementary Fig. 1A, B) and led to a dose-dependent increase in mROS in multiple PDAC cell lines tested (Fig. 1G, H). In line with these findings, we observed a dose-dependent loss of mitochondrial membrane potential (MMP) in PANC-1 and MIA PaCa-2 cells treated with AP-4-139B (Fig. 1I, J, Supplementary Fig. 1C, D). In sum, our results provide evidence that suppression of HSP70 impairs mitochondrial function in PDAC cells.

### HSP70 regulates mitochondrial subcellular localization and dynamics

**Fig. 2:**
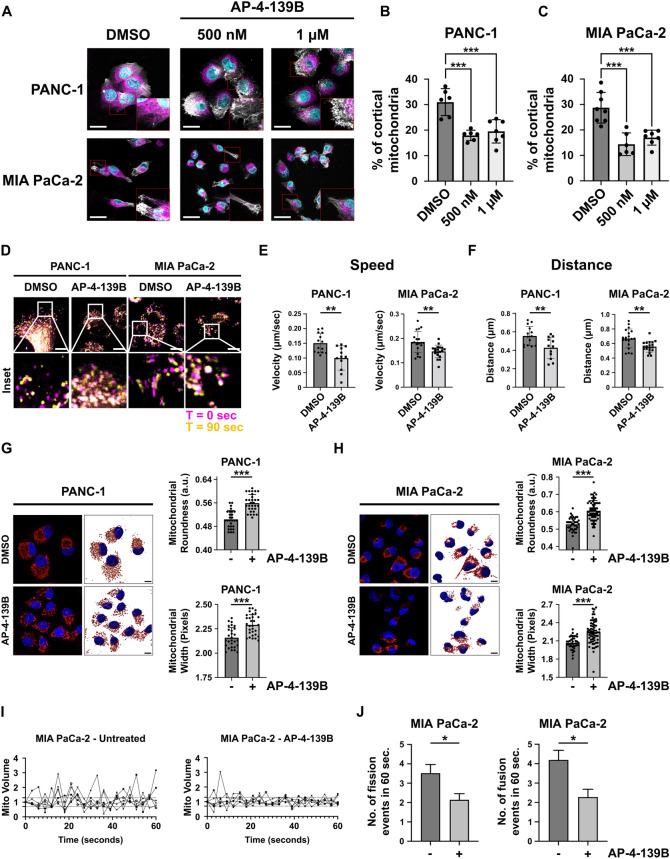
HSP70 inhibition affects mitochondrial subcellular localization and dynamics. **A** PANC-1 and MIA PaCa-2 cells were treated with 500 nM or 1 μM of AP-4-139B for 24 h and the percentage of cortical mitochondria were analyzed. Shown are representative images of mitochondria that were labeled with 100 nM MitoTracker deep red FM dye (magenta), while actin filaments were stained with phalloidin (white) and nuclei stained with Hoechst (cyan). Six to eight images were taken per experimental group via confocal microscopy at 40X magnification. Bar scale: 25 µm. **B**, **C** Quantification of (**A**); ***p < 0.001. **D** PANC-1 and MIA PaCa-2 cells were treated with 500 nM of AP-4-139B for 24 h and were analyzed for mitochondrial motility by time-lapse video-microscopy. Magenta = 0 seconds, yellow = 90 seconds, white = overlap. Bar scale: 10 µm. **E**, **F**. Quantification of (**D**); mitochondrial motility was measured and the speed (**E**) and the distance (**F**) of each mitochondrion were analyzed. 12-19 individual mitochondria were analyzed per treatment group for each cell line; **p < 0.01. **G**, **H**. PANC-1 (left) and MIA PaCa-2 (right) cells were treated with 2 μM AP-4-139B for 24 h, and were then stained with MitoTracker Red. Mitochondrial morphologies were 3D surfaced mapped using Imaris software. Bar scale: 10 µm; n = 2 biological replicates. To the right of each representative images are the quantification of mitochondrial roundness and width; ***p < 0.001. **I** MIA PaCa-2 cells were treated with 500 nM AP-4-139B for 24 h followed by staining with MitoTracker Deep Red. Time-lapse video-microscopy was performed by acquiring images every 3 s for a 1-min interval and change in mitochondrial volume over time was measured. n = 2 independent biological replicates, with six single cells imaged for each experimental condition. **J** Quantification of mitochondrial fission (<0.7-fold mitochondrial volume) and fusion (>1.3-fold mitochondrial volume) events in a 1-min time interval. *p < 0.05. Data are shown as mean ± SD.

Recent studies suggest that bioenergetically active mitochondria travel to the cortical cytoskeleton and localize to focal adhesion complexes to regulate pro-tumorigenic pathways, including enhanced cell motility and invasion of tumor cells [20]. To our knowledge, the impact of HSP70 on mitochondrial subcellular localization has never been studied. To test this, we plated PANC-1 and MIA PaCa-2 cells on coverslips in the presence or absence of AP-4-139B for 24 h. We then stained the cells with MitoTracker DeepRed followed by confocal microscopy and cortical mitochondrial analysis as previously described [21]. We found that HSP70 inhibition led to a redistribution of mitochondria from the peripheral/cortical cytoskeleton to the perinuclear region in both PDAC cell lines tested (Fig. 2A–C). Similar results were observed using pooled siRNA against the stress-induced HSP70 family member *HSPA1A* (Supplementary Fig. 1E–G), which localizes in part to the mitochondria of tumor cells [3]. Given that the functional role of HSP70 in mitochondrial dynamics has not been defined, we also wanted to determine whether HSP70 inhibition affects mitochondrial dynamics. Using time-lapse video-microscopy, we found that genetic or therapeutic ablation of HSP70 significantly impaired mitochondrial motility, as evidenced by a reduction in the speed of movement and a decrease in distance traveled by individual mitochondria in AP-4-139B treated PDAC cells (Fig. 2D–F, Supplementary Fig. 2A–C).

Mitochondrial subcellular distribution and motility are both influenced by mitochondrial structure, which is regulated by the processes of mitochondrial fission and fusion. These processes also regulate mitochondrial homeostasis and serve to maintain optimal mitochondrial function [22]. To test the impact of HSP70 inhibition on mitochondrial structure, we analyzed structural features of mitochondria in AP-4-139B treated PDAC cells stained with MitoTracker Red using Mito Hacker [23]. We observed significant differences in several structural features of the mitochondrial network upon HSP70 inhibition in both PANC-1 and MIA PaCa-2 cells, including mitochondrial roundness and width (Fig. 2G, H). Together, these findings are indicative of mitochondrial swelling, a key morphological feature of mitochondrial cell death [24]. These data are also consistent with a shift in the balance of fusion and fission activity [25]. To expand on these findings, we analyzed the mitochondria of PDAC cells by time-lapse video-microscopy and found that genetic ablation or pharmacological inhibition of HSP70 impaired mitochondrial networks in PANC-1 and MIA PaCa-2 cells, as demonstrated by a reduction in the rates of mitochondrial fission and fusion events (Fig. 2I, J, Supplementary Fig. 2D–I). Taken together, our results support the notion that HSP70 regulates mitochondrial dynamics and subcellular organelle trafficking, both of which are critical for the progression and metastasis of PDAC [16, 26]. Furthermore, induction of mitochondrial swelling by HSP70 inhibition supports the premise that suppression of HSP70 can promote the mitochondrial cell death program.

### Ablation of HSP70 suppresses the PINK1-mediated phosphorylation of DRP1

**Fig. 3:**
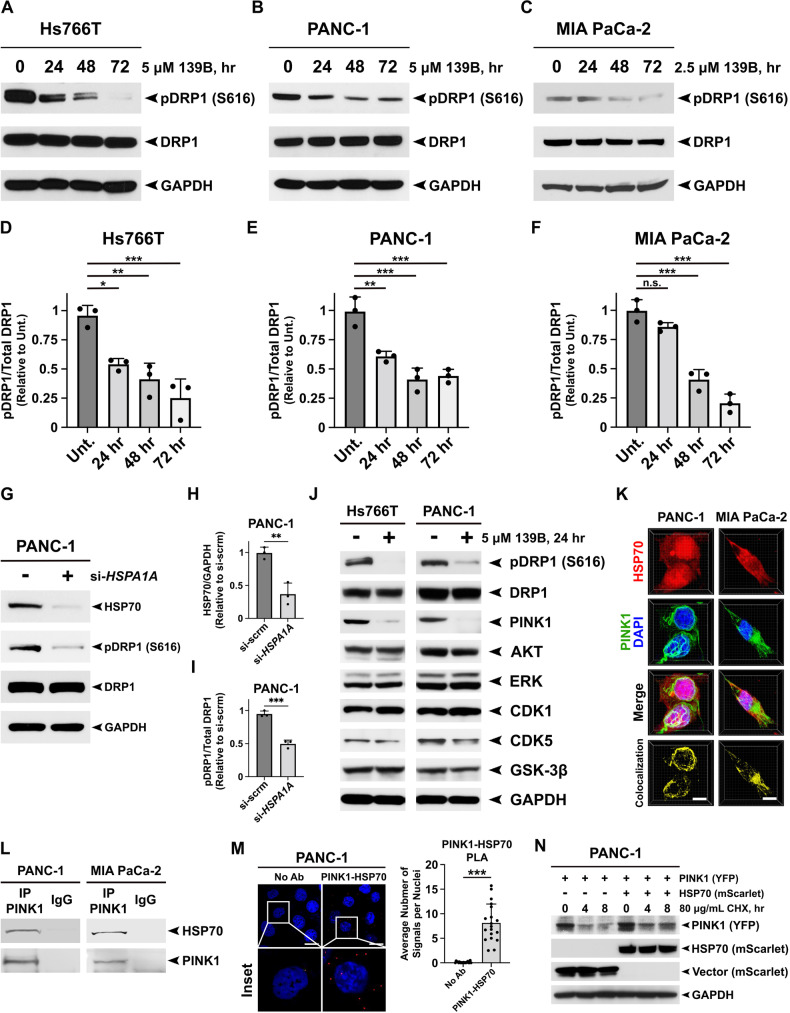
Genetic or therapeutic ablation of HSP70 impairs the PINK1-mediated phosphorylation of DRP1 at serine 616. **A**–**C** Hs766T, PANC-1, and MIA PaCa-2 cells were treated with the indicated doses of AP-4-139B and harvested every 24 h for 72 h. Cell lysates were subjected to Western blot analysis and immunoblotted for phospho-DRP1 (S616), total DRP1, and GAPDH (loading control). **D**–**F** Quantification of (**A**–**C**) was performed by obtaining the density of the phospho-DRP1 bands using ImageJ software and normalizing to total DRP1 levels. *p < 0.05, **p < 0.01, ***p < 0.001, n.s. not significant. Shown are quantification of three independent experiments. **G** PANC-1 cells were transfected with a pool of *HSPA1A* siRNA and were harvested 48 h later. Cell lysates were subjected to Western blot analysis and immunoblotted for phospho-DRP1 (S616), total DRP1, HSP70 and GAPDH (loading control). **H**, **I** Quantification of (**G**) was performed by obtaining the density of the phospho-DRP1 bands using ImageJ software and normalizing to total DRP1 levels. Quantification of HSP70 was normalized to GAPDH. **p < 0.01, ***p < 0.001. Shown are quantification of three independent experiments. **J** Hs766T and PANC-1 cells were treated with 5 μM AP-4-139B for 24 h. Cell lysates were subjected to Western blot analysis and immunoblotted for phospho-DRP1 (S616), DRP1, PINK1 (mature form), AKT, ERK, CDK1, CDK5, GSK-3β, and GAPDH (loading control). n = 3 independent experiments. **K** Co-immunofluorescence (Co-IF) analysis of PANC-1 and MIA PaCa-2 cells immunostained with HSP70 and PINK1, followed by fluorescent secondary staining along with DAPI (blue). Three-dimensional (3D) images were generated using Imaris imaging analysis software. n = 3 independent experiments. Bar scale: 10 µm. **L** Lysates from PANC-1 and MIA PaCa-2 cells were immunoprecipitated with IgG or anti-PINK1 antibodies and probed for HSP70. **M** Proximity Ligation Assays (PLA) for HSP70-PINK1 complexes in PANC-1 cells. Individual HSP70-PINK1 interactions are visualized by fluorescent signal (red) with nuclei counterstained with DAPI. Right; quantification of the HSP70-PINK1 interactions measured as the average number of PLA signals per nuclei from over 100 cells analyzed from random fields. n = 2 independent experiments. ***p < 0.001. Bar scale: 20 µm. **N** PANC-1 cells were transfected with a PINK1-YFP plasmid in the presence or absence of mScarlet-HSP70 plasmid for 48 h. Cells were then treated with 80 µg/mL of cycloheximide (CHX) and lysed at the indicated times following the addition of CHX. Protein levels of PINK1 and HSP70 were measured by Western blot analysis. GAPDH was used as a loading control.

DRP1 is a GTPase that promotes metabolic and mitochondrial changes necessary to promote pancreatic cancer [16, 25]. Therefore, we hypothesized that the impact of HSP70 inhibition on mitochondrial dynamics we describe herein may be dependent on DRP1. To test this, we first assessed the impact of HSP70 inhibition on DRP1 phosphorylation at serine 616, a post-translational modification that promotes mitochondrial fragmentation. We found that HSP70 inhibition with AP-4-139B significantly reduced the levels of phosphorylated DRP1 at serine 616 in three different PDAC cell lines tested (Fig. 3A–C). Quantification of Western blot analysis suggested a time-dependent decrease in DRP1-serine 616 phosphorylation upon HSP70 inhibition (Fig. 3D–F). We also observed a concurrent increase in the phosphorylation of DRP1 at serine 637 upon HSP70 inhibition in PDAC cells (Supplementary Fig. 3A). To experimentally recapitulate these results using genetic ablation of HSP70, we transfected PANC-1 and Hs776T cells with a pool of *HSPA1A* siRNA and assessed the phosphorylation of DRP1 at serine 616. Silencing of HSP70 led to markedly lower levels of DRP1 phosphorylation at serine 616 in both PDAC cell lines (Fig. 3G–I, Supplementary Fig. 3B–D). These data suggest that inhibition of HSP70 alters DRP1 phosphorylation, which may explain in part how HSP70 inhibition impairs mitochondrial dynamics.

We then sought to determine the mechanism by which HSP70 inhibition affects DRP1 phosphorylation to regulate mitochondrial dynamics in PDAC. The MAP kinase ERK2 has been shown to phosphorylate DRP1 at serine 616 to promote mitochondrial fission and pancreatic tumor growth [16]. Surprisingly, we found that PDAC cells treated with AP-4-139B did not show any significant changes in the phosphorylation or the total levels of ERK1/2 (Supplementary Fig. 3E–G), suggesting that HSP70i-mediated regulation of DRP1 is independent of ERK1/2. While there are several other protein kinases that can regulate the phosphorylation of DRP1 at serine 616, we next focused on kinases that (1) are classified as bona fide HSP70 client proteins and (2) have been implicated in DRP1-mediated mitochondrial dynamics. Of these, the serine/threonine protein kinase PTEN-induced kinase 1 (PINK1) has been reported to interact with HSP70 in overexpression studies [27, 28] and has been shown to phosphorylate DRP1 at serine 616 to regulate mitochondrial dynamics [29]. We found that PDAC cells treated with concentrations of AP-4-139B shown to reduce the phosphorylation of DRP1 at serine 616 also showed a significant reduction in the protein levels of PINK1 (Fig. 3J). Western blot analysis of additional kinases previously reported to regulate DRP1 phosphorylation at serine 616, including AKT [30], CDK1 [31], CDK5 [32] and GSK-3β [33], were not markedly affected by HSP70 inhibition (Fig. 3J).

Given that PINK1 is an intrinsically unstable protein that is subject to both cleavage and proteasomal degradation [34, 35], we sought to determine whether it might interact with, and be regulated by, HSP70. To test this, we first performed co-immunofluorescence (co-IF) of endogenous HSP70 and PINK1 in PANC-1 and MIA PaCa-2 cells. We found that both PDAC cell lines tested showed significant co-localization of PINK1 and HSP70, predominantly in the cytoplasm (Fig. 3K). Next, we assessed the ability of HSP70 to interact with PINK1 using two assays, co-immunoprecipitation (co-IP) and the proximity ligation assay (PLA). Immunoprecipitation with PINK1 antisera revealed HSP70 in the immunoprecipitated complexes of PANC-1 and MIA PaCa-2 cells (Fig. 3L). Furthermore, PLA performed in PANC-1 cells corroborated an interaction between HSP70 and PINK1 (Fig. 3M). Lastly, we co-expressed PINK1-YFP and mScarlet-HSP70 in PANC-1 cells and found that overexpression of HSP70 resulted in increased levels of PINK1 after treatment with cycloheximide (CHX) compared to mScarlet-Vector control (Fig. 3N). Taken together, our data support the premise that PINK1 is a bona fide client protein of HSP70 and support a novel mechanism by which HSP70 inhibition suppresses the PINK1-DRP1 axis to regulate mitochondrial dynamics in PDAC.

### HSP70 inhibition limits cancer cell migration in vitro and metastasis in vivo

**Fig. 4:**
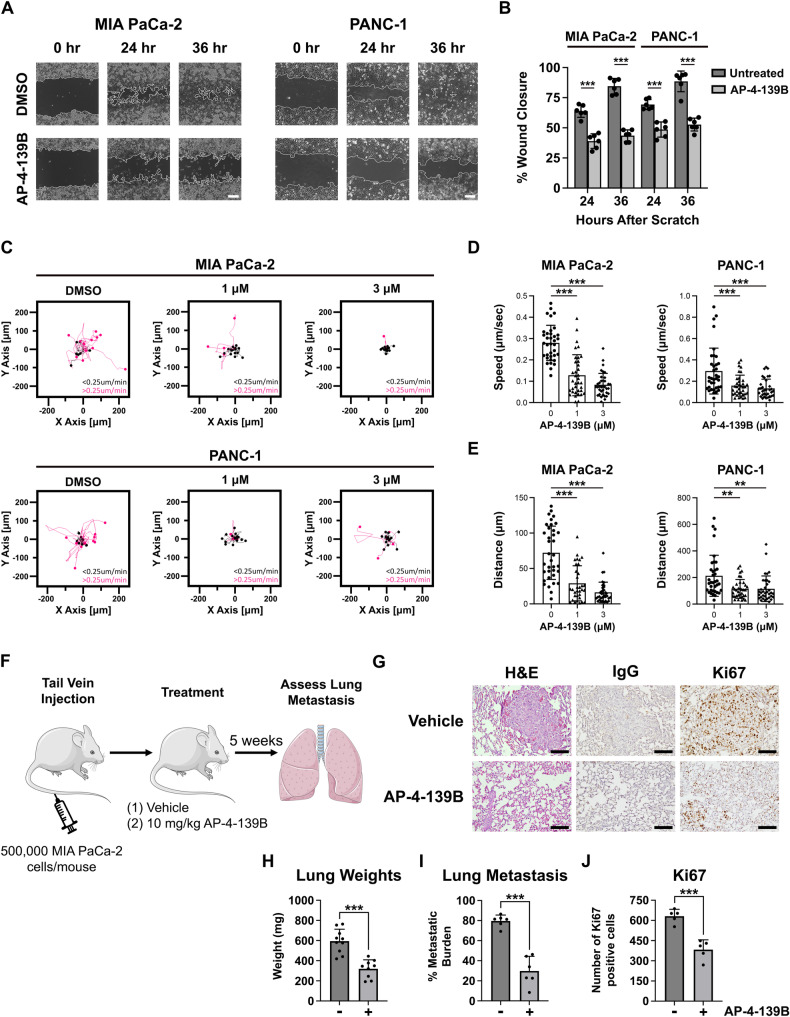
HSP70 inhibition limits PDAC cell migration in vitro and metastasis in vivo. **A** Primary (PANC-1) and metastatic (MIA PaCa-2) PDAC cells were seeded and allowed to form a confluent monolayer. Cells were scratched with a pipet tip and treated with DMSO or AP-4-139B and then imaged at 0, 24 and 36 h. Bar scale: 250 µm. **B** Quantification of the percentage of wound closure in (**A**) had 0, 24, and 36 h in DMSO versus AP-4-139B treated cells. The data depicted represent one representative containing data from three independent wells from a single experiment. n = 3 independent experiments; ***p < 0.001. **C** PANC-1 and MIA PaCa-2 cells were treated with the indicated doses of AP-4-139B for 24 h and were then analyzed for single-cell motility by time-lapse video-microscopy in 2D contour plots. The cutoff velocities for slow moving (black) or fast-moving (pink) cells are indicated. Quantification of the average speed of cell movements (**D**) and total distance traveled by individual cells (**E**). The mean ± SD speed of cell motility (µm/min), distance traveled (µm), and *p* values are indicated (n= approximately 35 cells per condition tested). **p < 0.01, ***p < 0.001. **F** Schematic representation of the metastasis assay. MIA PaCa-2 cells (5 × 10^5^) cells were injected into the tail vain of 8- to 10-week-old female NSG mice. Mice were treated with intraperitoneal (i.p.) injection of 10 mg/kg AP-4-139B every 48 h. After 5 weeks, the lungs of mice were formalin-fixed, and H&E stained and assessed for the presence of metastatic nodules. **G** Representative H&E and Ki67 images of lung metastases from NSG mice injected with MIA PaCa-2 cells in the tail vein, followed by treatment with vehicle or AP-4-139B. Bar scale: 100 µm. Rabbit IgG was used as a negative control for IHC analysis. Quantification of lung weights, metastatic burden, and Ki67 staining. Quantification of (**H**) was performed on all mice in the study, while quantification of (**I**, **J**) was performed on n = 5-6 mice per treatment group.

Given the role of DRP1 and mitochondrial dynamics in tumor-invasive phenotypes, we wanted to determine the impact of AP-4-139B on migration and metastasis in PDAC. For these studies, we used concentrations and timepoints of AP-4-139B that did not inhibit cell proliferation and were not cytotoxic when cells were grown as a monolayer. These assays revealed that after treatment with AP-4-139B, the ability of PANC-1 and MIA PaCa-2 cells to migrate was markedly impaired (Fig. 4A). By 36 h, the DMSO control groups were 90%+ filled in, whereas the AP-4-139B treated migration was approximately 50% (Fig. 4B). To further support these findings, we performed live-cell imaging on single cells to determine 2D tumor cell motility in response to HSP70 inhibition. Again, at doses that are non-cytotoxic, we noted significantly impaired motility of both PANC-1 and MIA PaCa-2 cells following treatment with AP-4-139B (Fig. 4C–E). Western blot analysis of these two cell lines showed that HSP70 inhibition led to a reduction in several markers of EMT, including Snail, Slug, and β-Catenin (Supplementary Fig. 3H, I), which may explain in part the decrease in the migration potential of PDAC cells treated with AP-4-139B.

We next sought to assess the ability of HSP70 inhibition to suppress pancreatic cancer metastasis in vivo. Toward this goal, we utilized the MIA PaCa-2 PDAC cell line, which is commonly used in metastasis assays because of its ability to colonize the lung following tail vein injection [36]. We injected MIA PaCa-2 cells into NSG mice via the tail vein, followed by intraperitoneal (i.p.) injections of 10 mg/kg AP-4-139B every other day (Fig. 4F). We found that treatment of mice with AP-4-139B led to a significant decrease in metastatic potential; this was accompanied by a decrease in lung weights and a decrease in Ki67 staining, a marker of proliferation, in lung metastases of mice treated with AP-4-139B (Fig. 4G–J). We found no obvious signs of toxicity at this dose, as there was no evidence of weight loss in AP-4-139B treated mice (Supplementary Fig. 3J, K). The combined data support the efficacy of AP-4-139B against metastatic PDAC in vivo.

### Single-agent efficacy of HSP70 inhibition in PDAC cells and xenograft models

**Fig. 5:**
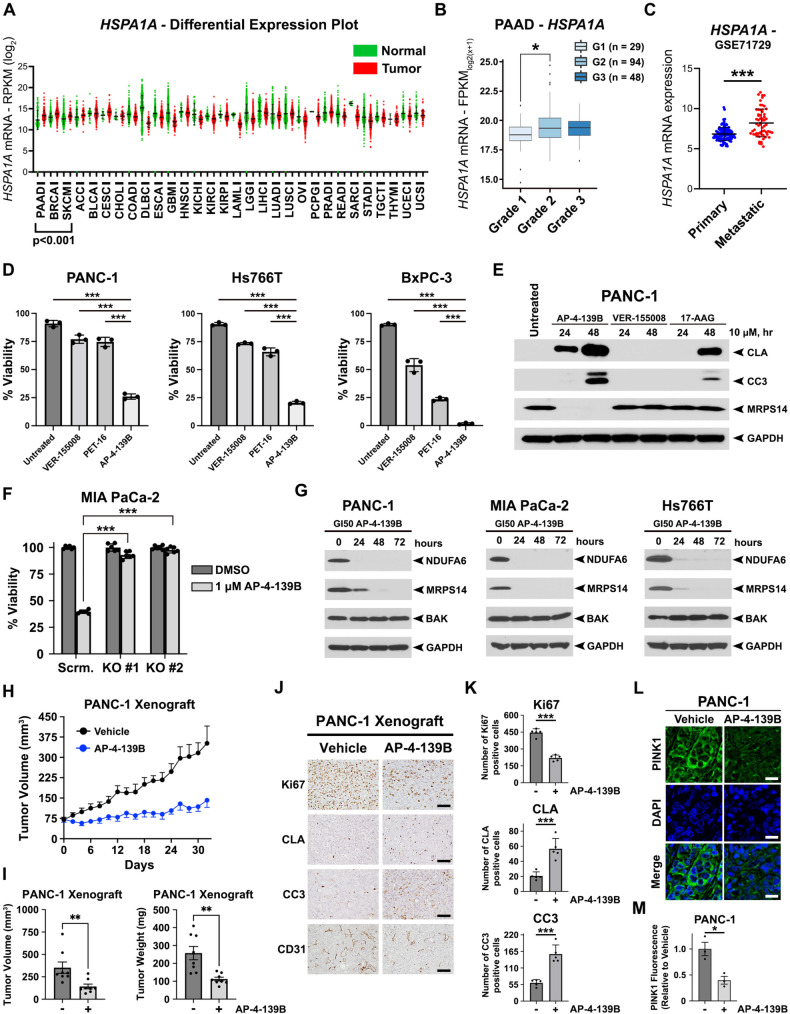
Single agent efficacy of AP-4-139B in PDAC cells and xenograft models. **A** Differential plots of *HSPA1A* between tumor and normal tissues of TCGA patients represented as reads per kilobase million (RPKM; log_2_) values. *P* < 0.001 where indicated. **B** Association of *HSPA1A* gene expression and tumor grade of pancreatic adenocarcinoma (PAAD; n = 171). ANOVA with post hoc Tukey pairwise comparison was used to determine significance. *p* = 0.017. **C** Association of *HSPA1A* gene expression in metastatic pancreatic cancer as compared to primary pancreatic tumors. ***p < 0.001. **D** PANC-1, Hs776, and BxPC-3 cells were treated with 10 µM of VER-155008, PET-16, or AP-4-139B for 48 h. Cells were then subjected to viability assays using trypan blue exclusion. ***p < 0.001. n = 3 independent experiments. **E** PANC-1 cells were treated with 10 µM of the indicated compounds and lysed at the indicated time points. Cell lysates were prepared for Western blot analysis and immunoblots were probed for CLA, CC3, MRPS14 and GAPDH (control). **F** Control (sg-scrambled) and two independent clones of HSP70 KO MIA PaCa-2 cells were treated with 1 µM AP-4-139B for 72 h and subjected to cell viability assays using Alamar Blue. Shown is a representative graph with six technical replicates per experimental group. n = 2 independent experiments. ***p < 0.001. **G** PANC-1, MIA PaCa-2, and Hs766T cells were treated with the respective GI_50_ values of AP-4-139B for the indicated time points. Cell lysates were subjected to Western blot analysis and immunoblotted for NDUFA6, MRPS14, BAK, and GAPDH (loading control). **H** PANC-1 cells were injected subcutaneously into the right flank of NSG mice. Once tumors reached an approximate volume of 75 mm^3^, mice were separated randomly into two groups. Mice were treated with either vehicle control or 10 mg/kg AP-4-139B every other day. Tumor volumes were measured over time using digital calipers. n = 8 mice per group; ***p < 0.001. **I** Quantification of tumor volume (left) and tumor weight (right) at endpoint; **p < 0.01. **J** IHC analysis of PANC-1 xenograft tumors treated with AP-4-139B. Shown are representative images (five random fields of view per condition) of Ki67, Cleaved Lamin A, Cleaved Caspase 3, and CD31. n = 5 mice per group. Bar scale: 100 µm. **K** Quantification of (**J**); ***p < 0.001. **L** Immunofluorescence analysis of PINK1 (green) was performed on PANC-1 xenograft tumors, counterstained with DAPI (blue). n = 3 mice per group. Bar scale: 20 µm. **M** Quantification of (**L**). Each data point represents an individual tumor, for which an average of six random images were taken and quantified. *p < 0.05.

To demonstrate the therapeutic potential and clinical relevance of targeting HSP70 in pancreatic cancer, we first analyzed the mRNA expression of HSP70 (*HSPA1A*) in different tumor types in comparison with their normal tissue counterparts using the Cancer Genome Atlas (TCGA) (https://www.cancer.gov/tcga). We found that HSP70 was significantly overexpressed in several different cancer types compared with normal tissues (Fig. 5A). More importantly, PDAC was the cancer type with the highest ratio of HSP70 in tumor versus normal tissue, highlighting the potential of HSP70 as a PDAC-specific target. We also found that HSP70 expression was highest in Grade II/III pancreatic cancer compared to Grade I tumors (Fig. 5B) and that expression was also increased in metastatic disease (Fig. 5C).

We next performed cell viability assays on a panel of human PDAC cell lines treated with three distinct HSP70 inhibitors: VER-155008, PET-16, and AP-4-139B. VER-155008 had little to modest ability to decrease PDAC cell viability and PET-16 showed effects on viability in a cell-type specific manner, whereas AP-4-139B was markedly more cytotoxic across all PDAC cell lines tested (Fig. 5D, Supplementary Fig. 3L, M). We extended these findings by performing Western blot analyses on five PDAC cell lines and found that AP-4-139B was the only HSP70 inhibitor able to induce cell death in all cell lines compared to other HSP70 inhibitors tested, as seen by an induction of multiple cell death markers including Cleaved Lamin A and Cleaved Caspase 3 (Supplementary Fig. 4A). At identical concentrations, AP-4-139B also showed superior efficacy in stimulating cell death in PDAC cells compared to the well-established HSP90 inhibitor 17-AAG (Fig. 5E). Next, we assessed the GI_50_ (growth inhibition – 50) of AP-4-139B in six PDAC cell lines of distinct genotypes, including cell lines with different *KRAS* and *TP53* mutations. We also compared the toxicity of AP-4-139B with other HSP70 inhibitors, as well as VY-3-277, which served as a negative control for AP-4-139B. VY-3-277 consists of the TPP group plus the linker but does not contain the planar group required to inhibit HSP70 [17]. As expected, VY-3-277 had GI_50_ values that were 15 to 60-fold higher than AP-4-139B. In addition, AP-4-139B performed in a superior manner compared to VER-155008 and PET-16 in all PDAC cell lines tested, irrespective of genotype (Supplementary Fig. 4B–E). To confirm that the effects of AP-4-139B were directly due to HSP70 inhibition, we generated two independent clones of HSP70 knockout (KO) MIA PaCa-2 cells using CRISPR/Cas9 technology (Supplementary Fig. 4F). We found that loss of HSP70 led to a significant increase in resistance to AP-4-139B in MIA PaCa-2 cells (Fig. 5F). Notably, protein levels of other HSP70 family members, including the cognate *HSPA8* (HSC70) and the mitochondrial *HSPA9* (GRP75), were unaffected in HSP70-KO cells (Supplementary Fig. 4F). Together, our data strongly support that AP-4-139B affects the viability of PDAC cells by specifically targeting the stress-induced HSP70.

We previously performed unbiased proteomic analysis on the mitochondria of AP-4-139B treated melanoma cells and identified novel HSP70 client proteins including MRPS14 and NDUFA6 [17]. Western blot analysis in three human PDAC cell lines treated with the GI_50_ of AP-4-139B showed significantly decreased levels of MRPS14 and NDUFA6 across all cell lines and time points tested, while the levels of non-HSP70 client proteins, such as BAK, were unaffected (Fig. 5G). Similar results were also found in several murine PDAC cell lines tested (Supplementary Fig. 4G), confirming the direct efficacy of AP-4-139B on the mitochondria of PDAC cells. Interestingly, VER-155008 and the HSP90 inhibitor 17-AAG did not cause a reduction in MRPS14 (Fig. 5E), consistent with the notion that HSP70 and HSP90 have some non-overlapping roles in proteostasis and cancer.

We then assessed the ability of AP-4-139B to impact tumor growth in vivo. Our data revealed that HSP70 inhibition significantly reduced the progression of both PANC-1 and MIA PaCa-2 tumor xenografts when mice were treated every other day with 10 mg/kg of AP-4-139B, compared to vehicle alone (Fig. 5H, Supplementary Fig. 5A); this was confirmed by tumor volumes and tumor weights at endpoint for both xenograft models (Fig. 5I, Supplementary Fig. 5B, C). We then analyzed tumor tissues using immunohistochemistry (IHC) and found that treatment with AP-4-139B led to reduced immunostaining for Ki67 (proliferation) and CD31 (angiogenesis), along with an increase in staining for cell death markers Cleaved Lamin A and Cleaved Caspase 3 (Fig. 5J, K, Supplementary Fig. 5D, E). We found no apparent signs of toxicity at this dose, as there was no evidence of weight loss or abnormal pancreas architecture (Supplementary Fig. 5F–J). Notably, we observed a significant reduction of PINK1 immunofluorescence in tumor tissues of PANC-1 and MIA PaCa-2 xenografts treated with AP-4-139B (Fig. 5L, M, Supplementary Fig. 5K, L). Taken together, the combined data support the premise that AP-4-139B is efficacious as a single agent to attenuate tumor progression in part by targeting mitochondrial function in vivo.

### Inhibition of HSP70 induces autophagic flux in pancreatic cancer cells

**Fig. 6:**
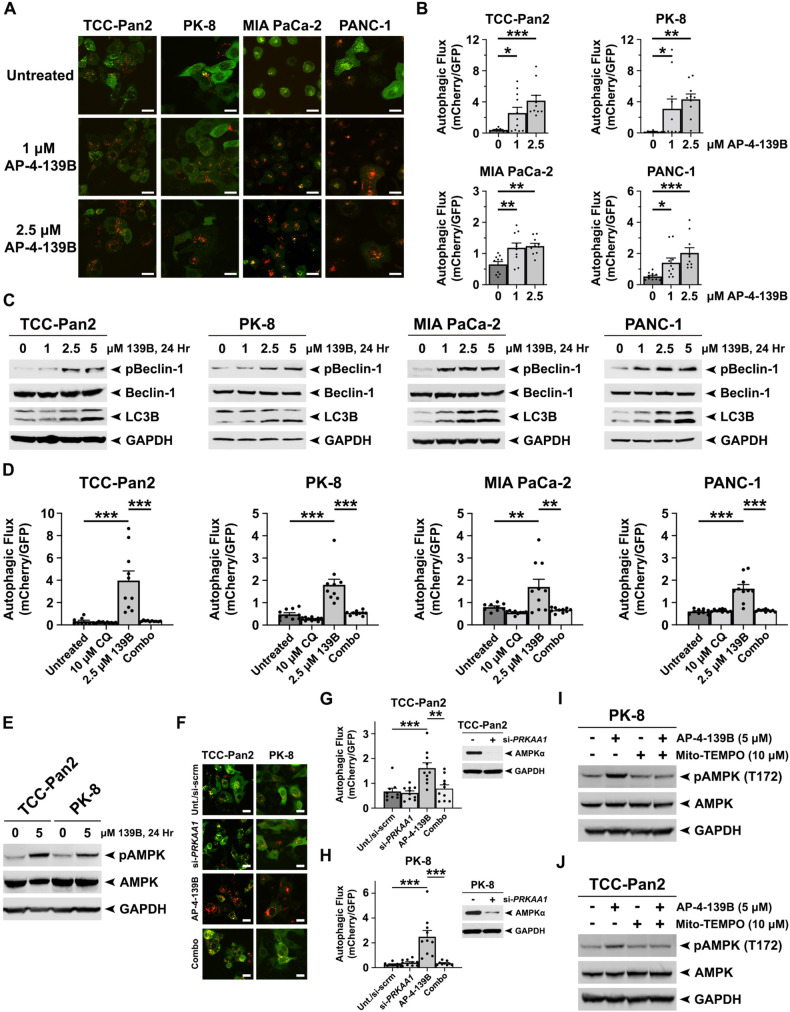
Pharmacological inhibition of HSP70 increases autophagic flux in PDAC cells. **A** Four PDAC cell lines (TCC Pan-2, PK-8, MIA PaCa-2, and PANC-1) were stably infected with a lentiviral vector encoding mCherry-EGFP-LC3B and then treated with the indicated doses of AP-4-139B for 6 h followed by confocal microscopy of EGFP+ and mCherry+ punctae. Shown are representative confocal images of merged EGFP and mCherry fluorescence that were used to quantify the autophagic index. Bar scale: 20 µm. **B** Quantification of (**A**). To quantify autophagic flux, the area ratios of mCherry-positive punctae to GFP-positive punctae (autophagic index) were determined. Mean autophagic index is plotted, with each individual data point representing one field of view (10 fields were analyzed per experimental group). n = 3 independent experiments. *p < 0.05, **p < 0.01, ***p < 0.001. **C** PDAC cell lines were treated with the indicated doses of AP-4-139B, or “139B,” for 24 h to assess autophagic flux. Immunoblot analysis of cell lysates were done to determine the levels of LC3B, phospho-Beclin-1 (S93), total Beclin-1, and GAPDH (loading control). Data are representative of three independent experiments. **D** PDAC cell lines were pre-treated with 10 µM of the autophagy inhibitor Chloroquine (CQ) overnight. The next morning, cells were treated with 2.5 µM AP-4-139B for 6 h, and then mean autophagic index was determined as in (**B**). Data are representative of two independent experiments, with each individual data point representing one field of view (9-10 fields were analyzed per experimental group). **p < 0.01, ***p < 0.001. **E**. TCC-Pan2 and PK-8 were treated with 5 µM AP-4-139B for 24 h. Immunoblot analysis of cell lysates were performed to determine the levels of phospho-AMPK (T172), total AMPK, and GAPDH (loading control). n = 2 independent experiments. **F** TCC-Pan2 and PK-8 stably expressing mCherry-EGFP-LC3B were transfected with a pool of *PRKAA1* siRNA. 48 h later, cells were treated with 2.5 µM AP-4-139B for 6 h followed by confocal microscopy of EGFP+ and mCherry+ punctae. Shown are representative confocal images of merged EGFP and mCherry fluorescence that were used to quantify the autophagic index. Bar scale: 20 µm. **G**, **H**. Quantification of (**F**) was performed as in (**B**), with each individual data point representing one field of view (9-10 fields were analyzed per experimental group). n = 2 independent experiments. **p < 0.01, ***p < 0.001. Shown to the right are Western blot analyses of AMPK and GAPDH (loading control). **I**, **J**. PK-8 and TCC-Pan2 cells were pre-treated with 10 µM of the mitochondrial ROS scavenger Mito-TEMPO for 3 h, at which point cells were subsequently treated with 5 µM AP-4-139B for 24 h. Cell lysates were subjected to Western blot analysis and probed for phospho-AMPK (T172), total AMPK, and GAPDH (loading control). Shown are representative results of two independent experiments.

Several reports have established that basal levels of autophagy are elevated in PDAC and may serve as a protective, anti-apoptotic mechanism against cytotoxic therapy [37–40]. However, the direct role of HSP70 in the regulation of autophagy in cancer remains somewhat controversial and may be due in part to tumor-specific differences across different studies performed. To determine whether HSP70 inhibition affects autophagy in PDAC, we used several biochemical techniques to investigate changes in autophagic flux upon inhibition of HSP70 [41]. First, we assessed autophagic flux in a panel of PDAC cell lines stably expressing the tandem fluorescence reporter mCherry-EGFP-LC3B. LC3B is an autophagy-related protein that undergoes post-translational modifications that lead to its lipidation and association with autophagic vesicles [42]. We found that HSP70 inhibition with AP-4-139B led to an increase in autophagic flux in four human PDAC cell lines tested (Fig. 6A, B). We then performed Western blot analysis to monitor the induction of the lipidated, autophagosome-associated LC3B-II as well as the phosphorylation of Beclin-1, a key driver of early-stage autophagy initiation and phagophore assembly [43]. We found that HSP70 inhibition significantly enhanced the levels of LC3B-II and activation of Beclin-1 as evidenced by enhanced phosphorylation at serine 93 (Fig. 6C). To expand on these findings and confirm the impact of HSP70 inhibition on autophagy, we performed additional autophagic flux assays in the presence or absence of chloroquine (CQ), an inhibitor of autophagy. As expected, treatment of PDAC cells with AP-4-139B caused a significant increase in autophagic flux; however, HSP70i-mediated induction of autophagy was significantly abrogated in the presence of CQ (Fig. 6D). Therefore, upon using multiple assays to monitor autophagy under different conditions in four human PDAC cell lines, our data support the premise that HSP70 inhibition leads to an induction of autophagic flux in PDAC.

AMPK is a critical energy sensor that is activated by phosphorylation [44]. Upon activation, AMPK can subsequently promote the phosphorylation of Beclin-1 to induce autophagy [45]. We found that PDAC cells treated with AP-4-139B display a significant induction in the phosphorylation of AMPK at threonine 172, a well-established marker of AMPK activation (Fig. 6E). Furthermore, we performed autophagic flux analysis of AP-4-139B treated PDAC cells in the presence or absence of pooled siRNA to AMPK (*PRKAA1*). These assays revealed that silencing of AMPK by siRNA suppressed the ability of AP-4-139B to induce autophagy in PDAC cells (Fig. 6F–H). While these data strengthen the role of AMPK in HSP70i-induced autophagy, the mechanism by which AMPK is activated by HSP70 inhibition is unknown. Recent reports have shown that AMPK is activated by the induction of mitochondrial ROS [46]. Given that HSP70 inhibition caused a significant induction of mitochondrial ROS in PDAC cells, we hypothesized that AMPK activation following HSP70 inhibition may be occurring via a similar mechanism. To test this, we pre-treated PK-8 and TCC-Pan2 cells with the mitochondrial-specific ROS scavenger Mito-TEMPO [47] for 3 h prior to HSP70 inhibition with AP-4-139B. Interestingly, we found that pre-treatment of PDAC cells with Mito-TEMPO significantly suppressed the ability of AP-4-139B to induce AMPK phosphorylation at threonine 172 (Fig. 6I, J). Taken together, these findings support the notion that HSP70 inhibition activates AMPK-mediated autophagy in part via the induction of mitochondrial ROS.

### Concurrent inhibition of HSP70 and autophagy impairs the progression of PDAC

**Fig. 7:**
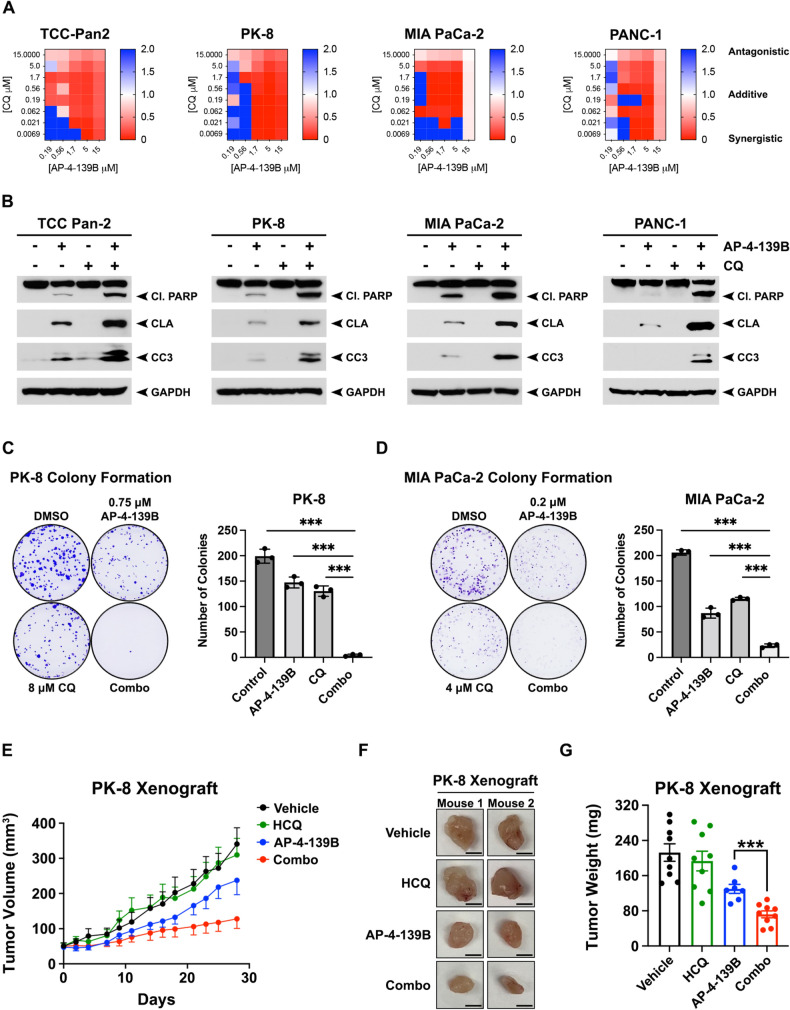
Dual inhibition of HSP70 and autophagy synergistically impairs the progression of PDAC. **A** A panel (n = 4) of PDAC cell lines were treated for 72 h with HSP70i (AP-4-139B) and the autophagy inhibitor CQ at the indicated concentrations. Cells were stained for viability with Calcein and imaged via Celigo image cytometer. Cell numbers at endpoint were normalized to vehicle-treated control (100% growth) for each cell line. Heatmaps representing BLISS independence scores corresponding using proliferation indices for each condition. Scores <1 indicate synergy (red), score = 1 indicated additivity (white), and scores >1 indicate antagonism (blue). n = 3 independent experiments. **B** PDAC cells (TCC Pan-2, PK-8, MIA PaCa-2) were treated with 5 µM AP-4-139B, 5 µM Chloroquine (CQ), or the combination of the two drugs for 48 h; PANC-1 cells were treated with 7.5 µM of AP-4-139B (139B) and CQ. Lysates were extracted and analyzed for Cleaved PARP (Cl. PARP), Cleaved Lamin A (CLA), and Cleaved Caspase 3 (CC3). GAPDH was used as a loading control. **C**, **D** PK-8 and MIA PaCa-2 cells were treated with the indicated doses of AP-4-139B, CQ, or the combination of AP-4-139B and CQ and subjected to colony formation assays. Seven (MIA PaCa-2) to fourteen (PK-8) days later, cells were fixed and stained with 0.1% Crystal Violet. Quantification of each assay is shown to the right of each representative figure. Values shown represent n = 3 for each treatment group ± the SD. *p < 0.05, **p < 0.01, ***p < 0.001. **E** 5 × 10^6^ PK-8 cells were subcutaneously injected into the flanks of NSG mice (n = 7–9 mice per group). Once tumors reached a size of approximately 50 mm^3^, mice were randomly assigned to each treatment group: Vehicle, AP-4-139B, Hydroxychloroquine (HCQ), or the combination (combo). Tumor growth was measured using digital calipers. **F** Images of representative tumors from PK-8 xenograft treatment groups in (**E**). **G** Quantification of PK-8 xenograft tumor weights at endpoint. *P* value is from two-sided, unpaired *t*-test comparing HSP70i treated to HSP70i plus HCQ treated tumors. Data are shown as mean ± SEM; sample sizes are as in (**E**).

We next sought to determine whether the combination of HSP70 and autophagy inhibition may be efficacious for the treatment of PDAC. Toward this end, we treated a panel of PDAC cell lines with AP-4-139B, CQ, or the combination of both inhibitors. BLISS synergy analysis revealed that the combination of AP-4-139B and CQ exhibited synergistic activity in all four PDAC cell lines tested (Fig. 7A). Given that CQ inhibits autophagy indirectly by affecting lysosomal function [48], we performed additional synergy assays using inhibitors that block specific components of the autophagic pathway, including Spautin-1 and MRT68921. Spautin-1 inhibits the ubiquitin-specific peptidases USP10 and USP13 that are necessary to initiate autophagosome formation [49]; MRT68921 targets the protein kinases ULK1/2, which are catalytic components of the autophagy pre-initiation complex [50]. Like CQ, Spautin-1 and MRT68921 synergized with AP-4-139B in all four PDAC cell lines tested (Supplementary Fig. 6A, B). We then performed Western blot analysis on the same four PDAC cell lines and found that treatment with the combination of AP-4-139B and CQ led to an induction of apoptosis, as evidenced by increased levels of Cleaved Lamin A, Cleaved Caspase 3, and Cleaved PARP compared with either agent alone (Fig. 7B). We then performed clonogenic survival assays and found that combining AP-4-139B with CQ significantly impaired colony formation in PK-8 and MIA PaCa-2 cells compared to either agent alone (Fig. 7C, D, Supplementary Fig. 6C, D). In sum, our data suggest that AP-4-139B synergizes with autophagy inhibitors to suppress the progression of PDAC in vitro.

We next evaluated the impact of concomitant HSP70 and autophagy inhibition on PDAC tumor growth in vivo. To test this, we subcutaneously injected PK-8 cells into the flanks of NSG mice. When tumors reached approximately 50 mm^3^, mice were randomized into four groups (*n* = 7–9 mice per group): Vehicle, AP-4-139B (10 mg/kg i.p. every other day), hydroxychloroquine (HCQ; 50 mg/kg/day i.p.), or the combination (AP-4-139B 10 mg/kg every other day; HCQ 50 mg/kg/day). Analysis of tumor growth revealed significant efficacy of AP-4-139B as a single agent against PK-8 tumors, while treatment with HCQ had essentially no impact on tumor growth. Notably, HCQ enhanced the efficacy of AP-4-139B in vivo, as demonstrated by a significant decrease in tumor volume with the combination therapy compared to AP-4-139B alone (Fig. 7E). This combination appears to be well tolerated, as we did not observe significant changes in the weights of these mice (Supplementary Fig. 6E, F). Additionally, tumors harvested from mice treated with the combination of AP-4-139B and HCQ were significantly smaller compared to tumors from mice treated with either AP-4-139B or HCQ alone (Fig. 7F, G). Collectively, our findings provide evidence that concurrent inhibition of HSP70 and autophagy may represent a novel therapeutic strategy to suppress the growth of PDAC.

## Discussion

Overexpression of HSP70 in tumor cells plays a cytoprotective role against proteotoxic stress and subsequent apoptosis [17]. In this study, we demonstrated that overexpression of HSP70 occurs in a subset of human cancers compared to their normal tissue counterparts using TCGA and other published datasets. Notably, our analyses identified PDAC as the cancer type with the highest ratio of HSP70 expression in tumor versus normal tissue. Beyond its canonical role in regulating proteostasis, we found that HSP70 inhibition altered mitochondrial subcellular localization and impaired mitochondrial motility in PDAC cells. Furthermore, mitochondria are dynamic organelles that undergo constant cycles of fission and fusion to maintain homeostasis, and this process has been shown to be dysregulated in human tumors. We found that HSP70 inhibition impaired mitochondrial dynamics and caused significant changes in mitochondrial structure. Similar results have been reported when treating KRAS mutant PDAC cells with ERK inhibitors [37]. This was due in part to a decrease in the phosphorylation in DRP1 at serine 616, an event that is essential for proper mitochondrial dynamics. These findings are consistent with previous reports showing that DRP1 phosphorylation at serine 616 is critical for KRAS driven tumor growth in a mouse model of PDAC [25]. In addition to dynamics, mitochondrial swelling is a key morphological feature observed during the activation of the mitochondrial cell death program; however, the mechanisms involved in the regulation of mitochondrial swelling are not fully understood. We show for the first time that HSP70 inhibition induced mitochondrial swelling in PDAC cells, supporting the premise that HSP70 inhibition can impair mitochondrial function and subsequently promote mitochondrial-mediated apoptosis.

While mitochondrial dynamics are typically viewed as antagonistic processes of fission and fusion events [51], recent studies have shown that the E3 ubiquitin ligase Parkin can suppress mitochondrial dynamics [52]. Functionally, re-introduction of Parkin in Parkin-negative cancer cells reduced the rates of both mitochondrial fusion and fission [52]. Herein, we demonstrate similar results in PDAC cells treated with AP-4-139B. These findings could be indicative of altered mitochondrial turnover, a process defined by a balance between mitochondrial biogenesis and mitophagy [53]. We envision at least two possibilities underlying the role of HSP70 in this process. The first is that HSP70 inhibition regulates mitochondrial dynamics in the absence of mitophagy. In support of this hypothesis, Parkin-mediated mitochondrial dynamics were shown to occur in a mitophagy independent manner [52]. Secondly, we propose that HSP70 inhibition may impact mitochondrial biogenesis. This may occur by regulating the PINK1-mediated phosphorylation and subsequent proteasomal degradation of the Parkin-interacting substrate PARIS, in turn stimulating mitochondrial biogenesis via peroxisome proliferator-activated receptor gamma coactivator-1 alpha (PGC-1α) [54]. These potential scenarios remain to be formally tested.

Clinical trials targeting heat shock proteins in cancer have shown varying levels of success. Over the last several decades, an abundance of HSP90 inhibitors have been discovered and entered clinical trials. However, these have shown limited clinical benefit due to drug resistance, dose-limiting toxicity, and poor pharmacokinetics [55, 56]. Given that HSP90 has been found to be essential for viability in all tested eukaryotes [57, 58], the observed gastrointestinal and liver toxicities are not surprising and have made the advancement of HSP90 inhibitors somewhat challenging. Unlike HSP90, HSP70 is not required for life and HSP70-knockout mice are viable and fertile [59], suggesting that targeting HSP70 in human cancers may show lower toxicity. In support of this premise, the HSP70 inhibitor Minnelide has demonstrated safety in Phase I trials and has advanced to Phase II in pancreatic cancer [60]. While Minnelide has been shown to suppress HSP70 [61], it can also target other critical cancer drivers including MYC [60]. These findings, along with other clinical trials using anti-tumor therapies concurrently with inhibitors of HSPs [62], suggest that tumor-specific combination strategies may significantly enhance the efficacy of HSP70 inhibitors.

Human PDAC exhibits elevated levels of basal autophagy, and recent reports have shown that suppression of RAS signaling in PDAC elicits an increase in autophagic flux, which was thought to serve as a protective mechanism for PDAC cells from RAS pathway inhibition [37, 63]. Consistent with these findings, we observed a similar phenotype when treating PDAC cells with the HSP70 inhibitor AP-4-139B, suggesting that PDAC cells may have an intrinsic desire to induce autophagy across multiple different classes of inhibitors. However, the respective contribution of HSP70i-mediated chaperone inhibition and mitochondrial toxicity to the induction of autophagy remains unclear and warrants further investigation. We also found that combining HSP70 inhibition with the autophagy inhibitor HCQ led to a substantial reduction in tumor growth in vivo. However, we must consider that autophagic flux in PDAC does not only occur within tumor cells, but also in cells within the PDAC TME. For example, autophagy in macrophages and stroma-associated pancreatic stellate cells (PSCs) plays a critical role in tumor maintenance [64, 65]. However, the impact of HSP70 inhibition on autophagy within the PDAC TME has not been investigated; we are currently pursuing this avenue.

Overall, our findings represent a novel therapeutic vulnerability in PDAC by targeting the mitochondrial fraction of HSP70. Unlike normal cells and tissues, cancer cells (including PDAC) have a significant fraction of the stress induced HSP70 that localizes to the mitochondria. Consequently, it became evident that new derivatives of HSP70 inhibitors for cancer therapy must consider the mitochondrial fraction of HSP70. Our data herein support this notion, in that AP-4-139B was markedly more cytotoxic against all PDAC cell lines tested relative to other established HSP70 inhibitors, including those that do not efficiently target mitochondrial HSP70. Notably, AP-4-139B effectively killed PDAC cells regardless of genotype or molecular phenotype and was efficacious as a single agent in primary and metastatic models of PDAC, without any apparent toxicity. Finally, our pre-clinical data provide a powerful rationale for testing the concurrent inhibition of HSP70 and autophagy in PDAC, and possibly other tumor types where autophagy may serve to protect cancer cells from cytotoxic therapy.

## Materials and methods

### Cell lines

PANC-1 and MIA PaCa-2 cell lines were a generous gift from Dr. Donna George (University of Pennsylvania, Philadelphia, PA, USA). Murine PDAC cells (2838.c3 and 6419.c5) were provided by Dr. Ben Stanger (University of Pennsylvania). Hs776T cells were provided by Dr. Rahul Shinde (The Wistar Institute, Philadelphia, PA, USA). PK-8 cells were from the Riken Cell Bank, and TCC-Pan2 cells were from the Japanese Collection of Research Bioresources Cell Bank [66]. BxPC-3, AsPC-1, PSN-1, and hTERT-HPNE cells were purchased from ATCC. Cells were grown in the media advised by ATCC, supplemented with 10% FBS (Cytiva, HyClone Laboratories, Logan, UT, USA), and 1% Gibco™ penicillin/streptomycin (15140-122; Thermo Fisher Scientific, Waltham, MA, USA) in a 5% CO_2_ humidified incubator at 37 °C. PK-8 and TCC-Pan2 cells were grown in the absence of Pen/Strep. Hs766T cells were also supplemented with Gibco^TM^ Insulin-Transferrin-Selenium (41400-045; Thermo Fisher Scientific). Cell lines were authenticated using short tandem repeat profiling and were tested for *Mycoplasma* every 6 months.

### Animal Studies

All studies were carried out in accordance with the recommendations in the Guide for the Care and Use of Laboratory Animals of the NIH. All protocols were approved by the Medical University of South Carolina (MUSC) Institutional Animal Care and Use Committee. Mice were housed in a temperature-controlled environment with *ad libitum* diet and maintained with a 12-h dark/12-h light cycle. At the end of all mouse studies, mice were euthanized, tissues were harvested and fixed in formalin for analysis. Body weight was measured either every other day or three times per week, and all mice were monitored daily for signs of pain or distress.

### Statistical analysis and other methods

Unless otherwise stated, all experiments were carried out with a minimum of three biological replicates (n = 3). All mouse experiments had n = 6–10 mice per experimental group. The log-rank test was used to analyze time-to tumor growth data and survival data. The Student *t* test or Wilcoxon rank-sum test was used for analyzing continuous variables. For in vitro studies, the two-tailed unpaired Student *t* test was performed for two-group comparisons. One-way ANOVA with either post hoc Holm-Šídák or post hoc Dunnett’s multiple comparisons test was used for multi-group comparisons. All in vitro data are reported as the mean ± standard deviation, unless stated otherwise, and all in vivo data are reported as the mean ± standard error. Statistical analyses were performed using GraphPad Prism software. *P* values are as indicated: *p < 0.05, **p < 0.01, ***p < 0.001; n.s. not statistically significant.

All other methods, including antibodies and reagents, Western blotting, generation of CRISPR knockout cell lines, immunohistochemistry (IHC), cell viability, colony formation assays, synergy assays, immunofluorescence (IF), co-immunoprecipitation (Co-IP), proximity ligation assays (PLA), plasmids, siRNAs, transfections, autophagic flux assays, mitochondrial oxygen consumption rates, mitochondrial depolarization assays, cortical mitochondria analysis, time-lapse video-microscopy, ROS production, tumor cell migration, motility, 2D chemotaxis, HSP70 gene expression analysis, and detailed animal studies are provided in the Supplementary Materials and Methods. The uncropped Western blots are provided in the Supplementary Materials.

### Supplementary information

Supplementary Materials

Uncropped Western blots

## Data Availability

All data needed to evaluate the conclusions on the manuscript are present in the paper and/or the Supplementary Materials. Such data, code, and materials are available to any researcher. TB is the corresponding author for this manuscript. AP-4-139B could be provided by M. Murphy and/or J. Salvino from The Wistar Institute, pending scientific review and a completed materials transfer agreement. Cell lines generously provided by researchers listed in the Materials and Methods may be contacted for such cell lines. Requests for these reagents should be submitted to these investigators. The GSE71729 dataset was used in this manuscript. The code generated to analyze TGCA PAAD data in Fig. 5B is available from the corresponding author upon request.
