# Supplementary material for: HSP70-mediated mitochondrial dynamics and autophagy represent a novel vulnerability in pancreatic cancer
Source: Cell Death Differ. 2024 May 28;31(7):881–96. doi: 10.1038/s41418-024-01310-9 (PMC11239841; doi:10.1038/s41418-024-01310-9)
Supplement: Supplementary file 1 — Supplementary Materials [file 41418_2024_1310_MOESM1_ESM.pdf]

SUPPLEMENTARY INFORMATION

HSP70-mediated mitochondrial dynamics and autophagy represent a novel vulnerability in pancreatic cancer

Giulia D. S. Ferretti[1,2,9], Colleen E. Quaas[1,2,9], Irene Bertolini[3], Alessandro Zuccotti[1,2], Ozge Saatci[1,2], Jennifer A. Kashatus[4], Salma Sharmin[4], David Y. Lu[3], Adi Narayana Reddy Poli[3], Abigail F. Quesnelle[1,2], Jezabel Rodriguez-Blanco[2,5], Aguirre A. de Cubas[2,6], G. Aaron Hobbs[2,7], Qin Liu[3], John P. O'Bryan[2,7,8], Joseph M. Salvino[3], David F. Kashatus[4], Ozgur Sahin[1,2] and Thibaut Barnoud[1,2*]

[1]Department of Biochemistry and Molecular Biology, Medical University of South Carolina, Charleston SC; [2]Hollings Cancer Center, Medical University of South Carolina, Charleston SC; [3]Molecular and Cellular Oncogenesis Program, The Wistar Institute, Philadelphia PA; [4]Department of Microbiology, Immunology, and Cancer Biology, University of Virginia Health System, Charlottesville VA; [5]Darby Children's Research Institute, Department of Pediatrics, Medical University of South Carolina, Charleston SC; [6]Department of Microbiology and Immunology, Medical University of South Carolina, Charleston SC; [7]Department of Cell and Molecular Pharmacology and Experimental Therapeutics, Medical University of South Carolina, Charleston SC; [8]Ralph H. Johnson VA Medical Center, Charleston SC.

Running Title: HSP70 regulates mitochondrial dynamics and autophagy

[9]G.D.S.F. and C.E.Q. contributed equally to this manuscript

Corresponding Author:
Thibaut Barnoud, PhD
Assistant Professor
Department of Biochemistry & Molecular Biology
Medical University of South Carolina, Hollings Cancer Center
86 Johnathan Lucas St, HO512E
Charleston, SC 29425
Phone: (843) 792-3535
Email: barnoud@musc.edu

Supplementary Materials and Methods

Antibodies and Reagents

Antibodies used were purchased from: GAPDH (2118S), Cleaved Caspase 3 (9661S), Cleaved Lamin A (2035S), HSP70 (4873S), ERK (4695T), AKT (9272S), GSK-3β (12456T), CDK5 (2506S), CDK1/cdc2 (9116T), Phospho-Beclin 1 (S93; 14717S), Beclin 1 (4122S), BAK (12105S), LC3B (3868T), PARP (9542S), Snail (3879T), Slug (9585T), β-Catenin (8480T), Ki-67 (9449S), CD31 (77699S), PINK1 (6946T), Phospho-DRP1 (S616; 4494S), and DRP1 (14647S), Phospho-DRP1 (S637; 6319S), β-Actin (5125S), phospho-AMPKα (2535T), AMPKα (5832T), phospho-ERK (4370T), and ERK (4695S) (Cell Signaling Technology, Danvers, MA, USA); MRPS14 (ab151118) (Abcam, Cambridge, MA, USA); PINK1 (23274-1-AP) (Proteintech, Rosemont, IL, USA); HSP70 (C92F3A-5) (Enzo Life Sciences, Farmingdale, NY, USA); HSC70 (sc-7298), GRP75 (sc-133137), and PINK1 (SC-518052) (Santa Cruz Biotechnology, Dallas, TX, USA); and NDUFA6 (GTX65550) (GeneTex, Irvine, CA, USA). The following fluorescent secondary antibodies were purchased from Thermo Fisher Scientific: Donkey anti-Mouse Alexa Fluor 488 (A-21202), Goat anti-Rabbit Alexa Fluor 647 (A-21244), Goat anti-Rabbit Alexa Fluor 488 (A-21206), and Goat anti-Rat Alexa Fluor 594 (A-48264). Mouse (7076S), rabbit (7074S), rat (7077S), and rabbit light-chain specific (93702S) secondary antibodies were purchased from Cell Signaling Technology. AP-4-139B was generously provided by Dr. Maureen Murphy (The Wistar Institute, Philadelphia, PA, USA). VER-155008 (HY-10941), 17-AAG (HY-10211), and Hydroxychloroquine (HY-B1370) were purchased from MedChemExpress (Monmouth Junction, NJ, USA). Spautin-1 (Item No. 17769) and MRT68921 (Item No. 19905) were purchased from Cayman Chemical (Ann Arbor, MI, USA). Chloroquine diphosphate salt (CQ; C6628) and Mito-TEMPO (SML0737-5MG) were purchased from MilliporeSigma (St. Louis, MO, USA).

Cycloheximide (CHX; J66901-03) was purchased from Thermo Fisher Scientific (Waltham, MA, USA). For *in vitro* studies, AP-4-139B, PET-16, and VER-155008 were dissolved in DMSO. CQ, CHX, and Mito-TEMPO were dissolved in molecular grade water.

Generation of HSP70 gene knockout cell lines

HSP70 knockout (KO) MIA PaCa-2 cells were generated by the Genome Engineering & Stem Cell Center (GESC@MGI) at the Washington University in Saint Louis. The guide RNA (gRNA) sequence for KO of *HSPA1A/1B* is 'atggccaaagccgcggcgat' followed by an NGG PAM site. A random guide sequence targeting no known human genes was used as a control. Briefly, knockout pools were nucleofected using a Lonza 4D nucleofection system (150,000 cells per 20 μL reaction) in P3 solution with program EH-100, with 1 μL WT spCas9 protein (Macrolabs) and 1 μL 100 μM sgRNA (obtained from Integrated DNA Technologies), recovered for 72 hours in 500 μL growth medium, then harvested for next generation sequencing (NGS) genotyping (Illumina sequencing) and expansion of pools. Clonal isolation of pools was performed using a Sony SH-800 cell sorter, genotypes were confirmed by NGS, and cell identify was confirmed by short tandem repeat (STR) profiling. All clones were confirmed as negative for mycoplasma contamination prior to cryopreservation.

Western blot analysis and Immunohistochemistry (IHC)

Western blot analyses and IHC were performed as described[1-4]. Briefly, $3x10^5$-$7.5x10^5$ cells were plated in 10cm tissue culture plates (10861-680; VWR International, Radnor, PA, USA) and treated as described. Cells were then harvested after the indicated timepoints and lysed with 1X RIPA buffer (89901; Thermo Fisher Scientific) supplemented with protease and phosphatase inhibitors

(11836170001 and 4906845001; purchased from MilliporeSigma). 25-100 µg of protein was run over SDS-PAGE gels using 10% NuPAGE Bis-Tris pre-cast gels (NP0301; Thermo Fisher Scientific) and were transferred onto polyvinylidene difluoride (PVDF) membranes (IPVH0010, pore size: 0.45 µm; MilliporeSigma). 15% SDS-polyacrylamide gels were used to obtain efficient separation and detection of LC3-I and LC3-II bands by Western blot. Following transfer, membranes were blocked using either 5% nonfat dry milk or 5% BSA (9999 or 9998, respectively; Cell Signaling Technology) for one hour at room temperature. Membranes were probed with indicated antibodies at 4°C overnight. Rabbit or mouse secondary antibodies conjugated to horseradish peroxidase (Cell Signaling Technology) were used at 1:5,000-1:20,000 dilutions and treated with Pierce ECL Western blotting substrate (Thermo Fisher Scientific) for two minutes. Protein levels were detected using autoradiography or the iBright CL1500 Imaging System (Thermo Fisher Scientific). Densitometry analysis of proteins was conducted using ImageJ software (NIH, Rockville, MD, USA). Paraffin embedding, tissue sectioning, and all IHC staining was performed by the Histology and Immunohistochemistry Laboratory Core at the Medical University of South Carolina. Images for IHC analysis were captured using a Leica DM2000 LED microscope, and the number of positive cells were quantified by ImageJ.

Cell viability, colony formation, and synergy assays

Alamar Blue assays were performed as previously described[5,6]. For Trypan Blue cell viability assays, $5x10^5$ PDAC cells were plated in 10 cm dishes. The next day, cells were treated with 10 µM of the indicated compounds for 48 hours. Cells were then collected and resuspended in a 1:1 ratio of 1X PBS and Trypan Blue (15259-061; Thermo Fisher Scientific). Live cell count and total cell count were determined manually using a hemocytometer. For colony formation assays, PK-8

and MIA PaCa-2 cells were seeded in 60 mm dishes (500 cells/dish) with the indicated concentrations of each compound, or the combination of both inhibitors. After 7 (Mia PaCa-2) and 14 days (PK-8), cells were fixed with 4% formaldehyde, stained with crystal violet solution (0.1% crystal violet in 10% ethanol), and washed abundantly with distilled water. Colony numbers were determined by manually counting using ImageJ cell counter plugin. Drug synergy assays were performed as previously described[7]. Briefly, 2000 cells/well of four PDAC cell lines (TCC-Pan2, PK-8, MIA PaCa-2, and PANC-1) were plated in 96-well plates. The next day, cells were treated with AP-4-139B in combination with one of three autophagy inhibitors (CQ, Spautin-1, or MRT68921) at the indicated concentrations for 72 hours. Total live cell count was quantified via Calcein staining (C3100MP; Thermo Fisher Scientific) and counted using a Celigo image cytometer. The average of 10 negative control wells (DMSO) was compared to a Day 0 control plate to determine base-line cell viability. Expected effect sizes for each treatment combination were calculated according to the BLISS algorithm[8]. Expected effect size was divided by observed effect size, and the results were then correlated to the corresponding heat maps where values >1 indicate antagonism (blue), =1 indicate additive (white), and <1 indicate synergy (red). All heat maps were generated using GraphPad Prism software; representative heatmaps shown were generated using average BLISS scores of two independent 96-well plates per experiment.

Immunofluorescence (IF)

For *in vitro* IF, PDAC cells were allowed to adhere overnight coverslips and subsequently fixed for 15 minutes (1% paraformaldehyde + 2% sucrose in 1X PBS), permeabilized (10 minutes in 1X PBS + 0.5% Triton X-100) and blocked for 30 minutes in the following solution: 1X PBS + 3% BSA + 0.2% Triton X-100. Cells were then incubated with primary antibodies PINK1 (Santa Cruz;

1:50) and HSP70 (Cell Signaling Technology; 1:100) for one hour (37ºC) and overnight (4ºC). Cells were then washed and incubated with the respective secondary antibodies (1:1,000). Nuclei were stained with DAPI (1 µg/mL) for 10 minutes in 1X PBS. After washing with PBS, coverslips were mounted with ProLong™ Diamond Antifade mounting medium (P36970; Thermo Fisher Scientific) and images were acquired on a Zeiss LSM 880 NLO microscope using a 63X oil objective with a 3.5X zoom. 3D images were generated using Imaris image analysis software. For *in vivo* IF, tumor tissues were fixed in 10% (V/V) formalin, embedded in paraffin, and sectioned by the Histology and Immunohistochemistry Core at the Medical University of South Carolina. Paraffin-embedded tissues were deparaffinized in 100% xylene and rehydrated in decreasing concentrations of ethanol. To eliminate fixation-caused autofluorescence, tissue sections were incubated in 10 mM Sodium Citrate (pH 6.0) in a water bath (95ºC) and cooled. Tissues were then permeabilized (30 minutes) and blocked for 2 hours with the same buffers described above, then incubated overnight with anti-PINK1 primary antibody (Proteintech – 1:400). Tissues were washed three times with 1X PBS and then incubated with Alexa Fluor™ 488 (1:500) secondary antibody. Slides were then washed 3 times, and nuclei were stained with DAPI (1 µg/mL) for 10 minutes in PBS and washed with distilled water to avoid PBS crystals. Coverslips were mounted and tissues imaged as described above for *in vitro* IF. The same parameters were used for acquiring all the images. Fluorescence intensity was measured using ImageJ Fiji software.

Co-immunoprecipitation (co-IP) and Proximity Ligation Assays (PLA)

PANC-1 and MIA PaCa-2 cells PDAC cells were allowed to adhere overnight. The next day, cells were harvested and centrifuged for 5 minutes at 4°C and washed with 1X PBS. Cell pellets were lysed in 300 µL of Pierce IP Lysis Buffer (87787; Thermo Fisher Scientific) with 1X Halt Protease

Phosphatase Inhibitor Cocktail (78440; Thermo Fisher Scientific) and incubated on ice for five minutes. Lysates were spun at 13,000 × g for ten minutes at 4°C. Protein extracts (4 mg) were incubated with either 10 μg of either PINK1 antibody (Cell Signaling Technology) or with Rabbit IgG (12-370; MilliporeSigma) for 1 hour at 4°C. HSP70 immunocomplexes were captured using Protein A magnetic beads (10-001-D; Thermo Fisher Scientific) and rotated overnight at 4°C. Beads were washed twice with lysis buffer, and 40 μL of 2× Laemmli Sample Buffer were added to the beads, and samples were boiled for 10 minutes at 95°C. PINK1/HSP70 association was analyzed by Western blot as described above. For PLA, PANC-1 cells were grown on Lab-Tek II 8-well chamber slides and fixed with 4% paraformaldehyde (15710; Electron Microscopy Sciences) followed by permeabilization with 0.25% Triton X-100 in 1X PBS (1132481001; MilliporeSigma). Protein–protein interactions were assessed using the PLA Duolink In Situ Starter Kit (DUO92101) (MilliporeSigma) according to the manufacturer's protocol, using the following primary antibodies: PINK1 (Proteintech; 1:400) and HSP70 (Enzo; 1:50). Nuclei were stained with DAPI (1 μg/mL) and the slides were mounted with ProLong™ Diamond Antifade mounting medium and images were captured on a Zeiss LSM 880 NLO microscope using a 63X oil objective. ImageJ Fiji software was used to quantify proximity ligation analyses (PLA) signals using the ImageJ cell counter plugin.

Plasmids, siRNA, transfections, and autophagic flux assays

PINK1-YFP (101874), mScarlet-HSP70 (163790), and mScarlet-Vector (85042) plasmids were purchased from Addgene (Watertown, MA, USA). For transient transfections, $1x10^6$ PANC-1 cells were plated in 10 cm dishes. The next day, 5μg of each of the indicated plasmids were transfected into cells using FuGENE 6 purchased from Promega (E2691; Madison, WI, USA) according to

the manufacturer's instructions. 48 hours later, PANC-1 cells were treated with 80µg/mL of cycloheximide (CHX) and harvested at the indicated timepoints for Western Blot analysis. For siRNA studies, Dharmacon™ scrambled control siRNA (D-001810-10-20), human *HSPA1A* siRNA (L-005168-00-0010), and human *PRKAA1* siRNA (L-005027-00-0010) were purchased from Revvity (Waltham, MA, USA). For siRNA transfections, $3x10^5$ PDAC cells were plated in 10-cm plates. siRNAs were transfected using Lipofectamine™ RNAiMAX transfection reagent (13778-075; Thermo Fisher Scientific) in OPTI-MEM™ media (31985-070; Thermo Fisher Scientific) according to the manufacturer's protocol. Cells were harvested 48 hours later for Western blot analysis. Autophagic flux assays were performed as previously described[9]. Briefly, PDAC cell lines were transduced with a pBABE-puro retroviral plasmid containing a mCherry-EGFP-LC3B construct (22418; Addgene, Watertown, MA, USA). $1x10^5$ PDAC cells per quadrant were plated on glass-bottomed plates (627870; Greiner Bio-One, Monroe, NC, USA) and treated with vehicle or AP-4-139B for six hours at indicated concentrations. For concurrent treatment with chloroquine (CQ), cells were pre-treated with CQ at the indicated concentrations overnight, followed by treatment with AP-4-139B the next day for six hours. Cells were imaged using a Zeiss 510 LSM confocal microscope. To determine levels of autophagic flux, the ratio of total area florescence of mCherry positive punctae and EGFP positive punctae was analyzed by ImageJ software as described previously[10].

Mitochondrial oxygen consumption rates (OCR), Mito-TEMPO, and mitochondrial depolarization assays

Mitochondrial Oxygen Consumption Rates using the XF Mito Stress Test was performed as previously described[11,12]. $2x10^4$ PANC-1 and HPNE-DT cells, and $5x10^4$ MIA PaCa-2 cells, were plated in Seahorse 96-well cell culture microplates, treated with AP-4-139B for 24 hours, and

subjected to the Seahorse XF Cell Mito Stress Test (103015-100), according to the manufacturer's protocol (Agilent Technologies, Santa Clara, CA, USA). Briefly, cell medium was replaced with Seahorse XF Base Medium (supplemented with 100 mM Pyruvate, 200 mM Glutamine, and 2.5 M Glucose) and incubated in a 37°C non-CO_2 incubator for one hour before the start of the assay. Basal OCR was measured using the Seahorse XFe Extracellular Flux analyzer. Measurements were performed after injection of three compounds affecting bioenergetics: 1 μM oligomycin, 1 μM carbonyl cyanide 4-(trifluoromethoxy) phenylhydrazone (FCCP), and 1μM Rotenone/Antimycin A; all inhibitors were purchased from Seahorse Bioscience. Data are representative of three biological replicates. For experiments performed in the presence or absence of Mito-TEMPO, PDAC cells were pre-treated for three hours with 10μM Mito-TEMPO, and subsequently treated with 5 μM AP-4-139B for another 24 hours. Cells were then harvested and processed for Western Blot analysis. For mitochondrial membrane depolarization assays, PANC-1 or MIA PaCa-2 cells were plated (2000 cells/well) in a 96-well plate and treated the following day with the indicated doses of AP-4-139B for 24 hours. Cells were incubated with 50 nM TMRE (T-669; Thermo Fisher Scientific) for 30 minutes at 37°C, followed by washing twice with 100 μL of 1X PBS. A final volume of 100 μL of PBS was added to each well, and the fluorescence was measured with the following excitation/emission: 544/590 nm. For TMRE measurements via microscopy, $1x10^5$ PDAC cells were plated per quadrant on glass-bottomed plates (627870; Greiner Bio-One) and treated with the indicated doses of AP-4-139B for 24 hours. Cells were then stained with TMRE for 20 minutes at 37°C, washed twice with 1X PBS, and nuclei were stained with two drops of Hoechst 33342 purchased from Thermo Fisher Scientific (R37605) and immediately imaged on a Zeiss LSM 880 NLO microscope using a 63X oil objective. ImageJ Fiji software was used to quantify the Intensity of TMRE fluorescence.

Cortical mitochondrial analysis, time-lapse video-microscopy, and ROS production

For cortical mitochondrial analysis, $1x10^4$ MIA PaCa-2 and PANC-1 cells were seeded on coverslips and treated with AP-4-139B (500nM and 1μM, respectively) for 24 hours. Cells were stained with 100 nM MitoTracker deep red FM dye for 30 minutes at 37°C, washed, and fixed with 4% formaldehyde for ten minutes. Actin filaments were stained with phalloidin 488 (Thermo Fisher Scientific) for one hour, and nuclei were stained with Hoechst (Thermo Fisher Scientific) for five minutes. Slides were analyzed on a Leica TCS SP5 confocal laser microscope with a 63X oil objective and images were analyzed in ImageJ as described[13]. For time-lapse video-microscopy, $1x10^4$ MIA PaCa-2 and PANC-1 cells were seeded on high-optical-quality glass-bottom 35-mm plates (MatTek Corporation) for 24 hours and then treated with 500nM or 1μM of AP-4-139B, respectively. Cells were then stained with MitoTracker Deep Red for 30 minutes at 37°C. Cells were imaged on a Leica TCS SP8 X inverted confocal laser scanning microscope using a 63X, 1.40-numerical-aperture (NA) oil objective, and live imaging time-lapse video-microscopy was performed using a Tokai Hit incubation chamber equilibrated to bidirectional scanning at 8,000 Hz at 37°C with 5% CO_2. Images were acquired every 3 seconds for a 1-minute interval. Individual 12-bit images were acquired using a white-light supercontinuum laser (0.2% at 643 nm) and hybrid detectors at a 4X digital zoom with a pixel size of 70 nm by 70 nm and a step size of 0.260 μm. A minimum of six single cells under each condition were collected for analysis. Initial postprocessing of 3D sequences was carried out as described[14]. Images were then imported into Leica LAS X software to either measure mitochondrial motility or mitochondrial fission and fusion events as described[14]. To detect intracellular ROS, MIA PaCa-2 and PANC-1 cells were treated with the indicated doses of AP-4-139B for 48 hours and then incubated with either CellROX-Green (C10444; Thermo Fisher Scientific) or MitoSOX-Red (M36008; Thermo

Fisher Scientific), according to the manufacturer's instructions. Cells were harvested and analyzed on a BD Biosciences FACSCelesta flow cytometer and intact cells were gated in the FSC/SSC plot to exclude small debris. The resulting data were plotted on a histogram. For analysis of mitochondrial morphology, 1,000 PANC-1 and MIA PaCa-2 cells were seeded on glass coverslips and treated with AP-4-139B (2 μM) for 24 hours. Cells were then treated with 50 nM MitoTracker Red CMXRos (Thermo Fisher Scientific) for 30 minutes, fixed, permeabilized, and mounted immediately using Prolong Gold antifade reagent with DAPI (P36935; Thermo Fisher Scientific). Cells were imaged on a Zeiss LSM 900 Airyscan2 microscope using a 63X oil objective, and mitochondrial morphologies were 3D surfaced mapped using Imaris microscopy image analysis software.

Tumor cell migration, motility and 2D chemotaxis

Scratch assays were performed as previously described[15]. Briefly, MIA PaCa-2 and PANC-1 cells were each seeded in 6-well plates and grown to form a confluent monolayer. Subsequently, two linear scratches, oriented perpendicular to each other, were made in each well with sterile 200 μL pipette tips, and wells were then washed twice with PBS. DMSO was used as an untreated control, and media with AP-4-139B at the indicated concentrations were added to the respective wells before beginning time-lapse imaging with a Nikon Te300 Inverted Microscope (3 images/well, 2 wells/condition). Images were captured every four hours for 36 hours. Cell migration was evaluated at 24- and 36-hour timepoints using ImageJ software (National Institutes of Health, USA) and normalized to wounds at the 0-hour timepoint. Results are expressed as the percent wound closure for each condition. For 2D motility studies, MIA PaCa-2 and PANC-1 cells ($1x10^4$) were seeded in 4-well Ph+ chambers (80446; Ibidi USA, Inc., Fitchburg, WI, USA) in complete

medium. Cells were treated with the indicated doses of AP-4-139B for 24 hours. Time-lapse video-microscopy was then performed over a 10-hour interval as described.[16]. Stacks were imported into ImageJ Fiji software for analysis, and 30 cells per each condition tested were tracked using the Manual Tracking plugin for ImageJ Fiji in 3 independent experiments. Tracking data were exported into Chemotaxis and Migration Tool v. 2.0 (Ibidi USA, Inc.) for graphing and calculation of means of speed and accumulated distance of movement.

HSP70 Gene Expression Analysis

The comparison of HSPA1A mRNA expression in normal, tumor, and metastatic site was performed as previously described[17-19]. Briefly, the mRNA expression of *HSPA1A* in TCGA tumors and matched normal tissue and in normal tissue from GTEx Biobank was retrieved using the Xena Browser[20], USCS Santa Cruz (https://xenabrowser.net/). The data were represented as scatter plots with mean and standard deviation using GraphPad Prism. The significance between tumor vs. normal expression of *HSPA1A* was calculated by unpaired students' t-test. The three cancer types where *HSPA1A* expression is significantly higher in tumors compared to normal (P<0.001) were ordered with respect to the difference between tumor and normal means from highest to lowest (i.e., PAAD, BRCA and SKCM). All the other cancer types were alphabetically ordered. To compare *HSPA1A* mRNA expression between primary pancreatic tumors and metastatic site, data from GSE71729 dataset[21] were retrieved from the GEO depository (https://www.ncbi.nlm.nih.gov/geo/) and represented as a scatter plot with mean and standard deviation using GraphPad Prism. The significance was calculated by unpaired students' t-test. For association of HSP70 expression and tumor grade, PAAD (pancreatic adenocarcinoma) patient data from TCGA were stratified into three groups: Grade I, II, and III. Data are represented as

boxplots using the median expression for *HSPA1A* across three grades. Significance between groups was determined using ANOVA with post hoc Tukey pairwise comparison. Boxplots were generated using ggplot R package, and analysis was completed using RStudio software.

Animal Studies

For human xenograft studies, 2.5×10^6 PANC-1 or MIA PaCa-2 cells were injected subcutaneously into the right flanks of 6–8-week-old female NSG (NOD.Cg-*Prkdc*^{scid} *Il2rg*^{tm1Wjl}/SzJ) mice; all mice were purchased from the Jackson Laboratory. For PK-8 xenograft studies, 5×10^6 cells were injected per mouse. Tumor volumes were measured using digital calipers and calculated using the formula: volume = (length x width^2) x 0.52. AP-4-139B was made as a stock solution of 100 mg/mL in DMSO and diluted to 2 mg/mL in 0.9% saline solution (S8776; MilliporeSigma), and Hydroxychloroquine (HCQ) was made as a 2mg/mL stock in PBS. Mice were given either vehicle control or 10mg/kg AP-4-139B every other day by intraperitoneal (i.p.) injection. For synergy studies, HCQ was given at 50mg/kg every day by i.p. injection. For *in vivo* metastasis assays, 8–10-week-old NSG mice were injected with 5×10^5 MIA PaCa-2 cells. Mice were then treated with either vehicle or 10 mg/kg AP-4-139B by i.p. injection every 48 hours. There was no blinding during *in vivo* experiments. Sample sizes were based on previous studies [22].

Supplementary Figures

Supplemental Figure 1

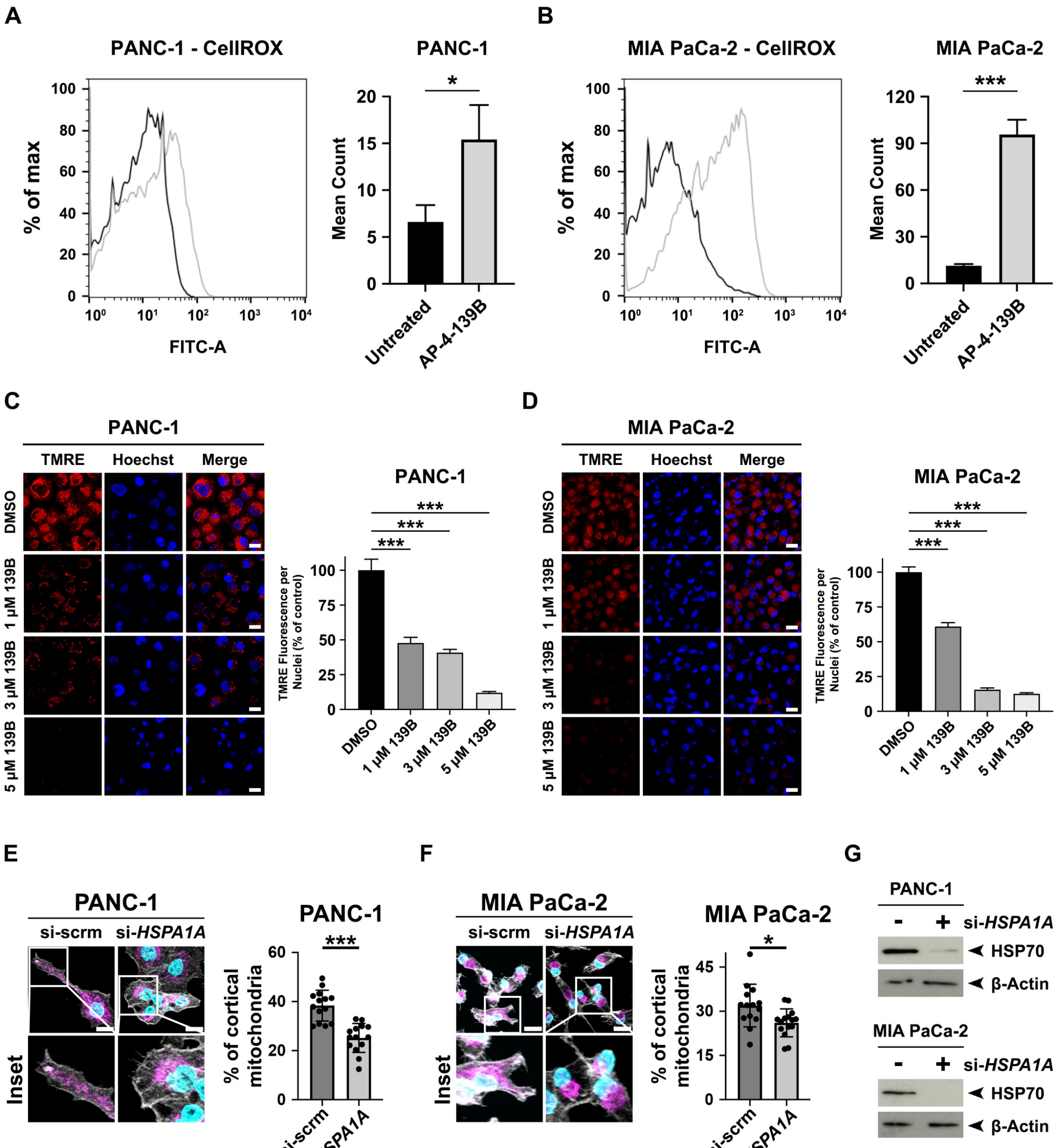

Supplemental Figure 1. Suppression of HSP70 impairs mitochondrial function and alters mitochondrial subcellular localization in PDAC cells.

A-B. PANC1 (A) and MIA PaCa-2 (B) cells were treated with 5 μM of AP-4-139B for 48 hours. Cells were then incubated with CellROX-Green, harvested, and analyzed by flow cytometry to determine ROS production. Fluorescence mean was analyzed and plotted on a histogram. To the right of each histogram is the quantification of mean PANC1 (A) and MIA PaCa-2 (B) CellROX counts in untreated and AP-4-139B treated cells. $*p<0.05$, $***p<0.001$; n=3 independent experiments.

C-D. PANC-1 and MIA PaCa-2 cells were treated with the indicated doses of AP-4-139B for 24 hours and were stained with TMRE for 20 minutes prior to imaging using a confocal microscope. Hoechst was used to stain nuclei. n=2 independent experiments. For each experiment, six random images were taken and quantified per experimental group. $***p<0.001$; bar scale: 20 μm.

E-F. PANC-1 and MIA PaCa-2 cells were transfected with a pool of *HSPA1A* siRNA for 48 hours and the percentage of cortical mitochondria were analyzed. Shown are representative images of mitochondria were labeled with 100 nM MitoTracker deep red FM dye (magenta), while actin filaments were stained with phalloidin (white) and nuclei stained with Hoechst (cyan). n=2 independent experiments. Six to eight images were taken per experimental group via confocal microscopy at 40X magnification. $*p<0.05$, $***p<0.001$; bar scale: 20 μm.

G. Western blot analysis of HSP70 protein levels following transfection of *HSPA1A* siRNA; GAPDH was used as a loading control.

Supplemental Figure 2

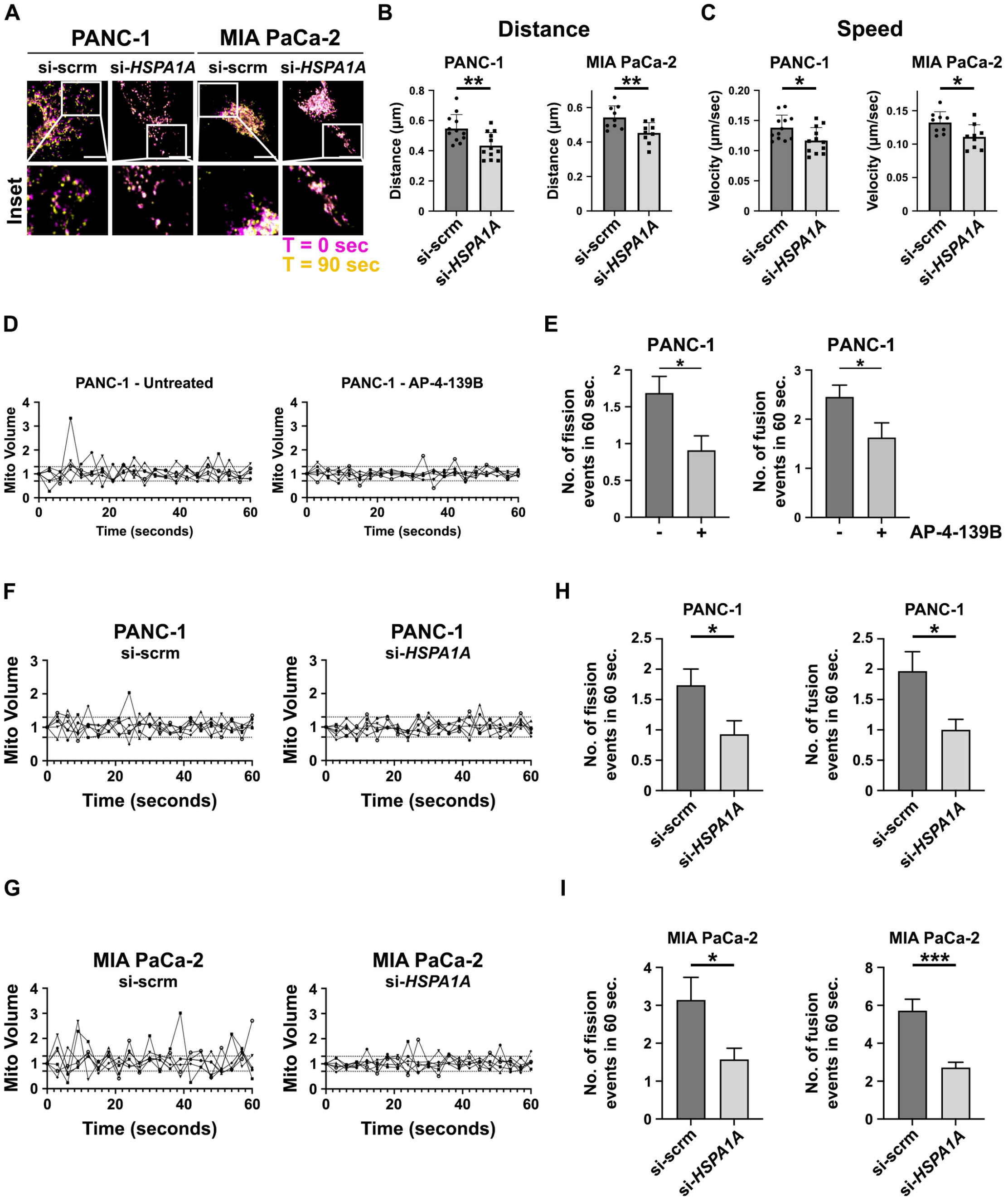

Supplemental Figure 2. Genetic or pharmacological ablation of HSP70 reduces mitochondrial motility and impairs mitochondrial dynamics in PDAC cells.

A. PANC1 and MIA PaCa-2 cells were transfected with a pool of *HSPA1A* siRNA for 48 hours and were analyzed for mitochondrial motility by time-lapse video-microscopy. Magenta = 0 seconds, yellow = 90 seconds, white = overlap; bar scale: 10 μm.

B-C. Quantification of (A); mitochondrial motility was measured and the distance (B) and the speed (C) of each mitochondrion were analyzed. 9-12 individual mitochondria were analyzed per treatment group for each cell line. n=2 independent experiments.

D. PANC-1 cells were treated with 500 nM AP-4-139B for 24hours, followed by staining with MitoTracker Deep Red. Time-lapse video-microscopy was performed by acquiring images every three seconds for 1-minute interval and change in mitochondrial volume over time was measured. n=2 independent biological replicates, with six single cells imaged for each experimental condition.

E. Quantification of mitochondrial fission (<0.7-fold mitochondrial volume) and fusion (>1.3-fold mitochondrial volume) events in a 1-minute time interval. $p<0.05$. Data are shown as mean ± SD.

F-G. PANC-1 and MIA PaCa-2 cells were transfected with *HSPA1A* siRNA for 48 hours followed by staining with MitoTracker Deep Red. Time-lapse video-microscopy was performed by acquiring images every three seconds for a 1-minute interval and change in mitochondrial volume over time was measured. Line graphs of (F) and (G) are of a single representative experiment; n=three independent biological replicates.

H-I. Quantification of mitochondrial fission (<0.7-fold mitochondrial volume) and fusion (>1.3-fold mitochondrial volume) events in a 1-minute time interval. Graphs of (G) and (I) are the average of three independent experiments.

Supplemental Figure 3

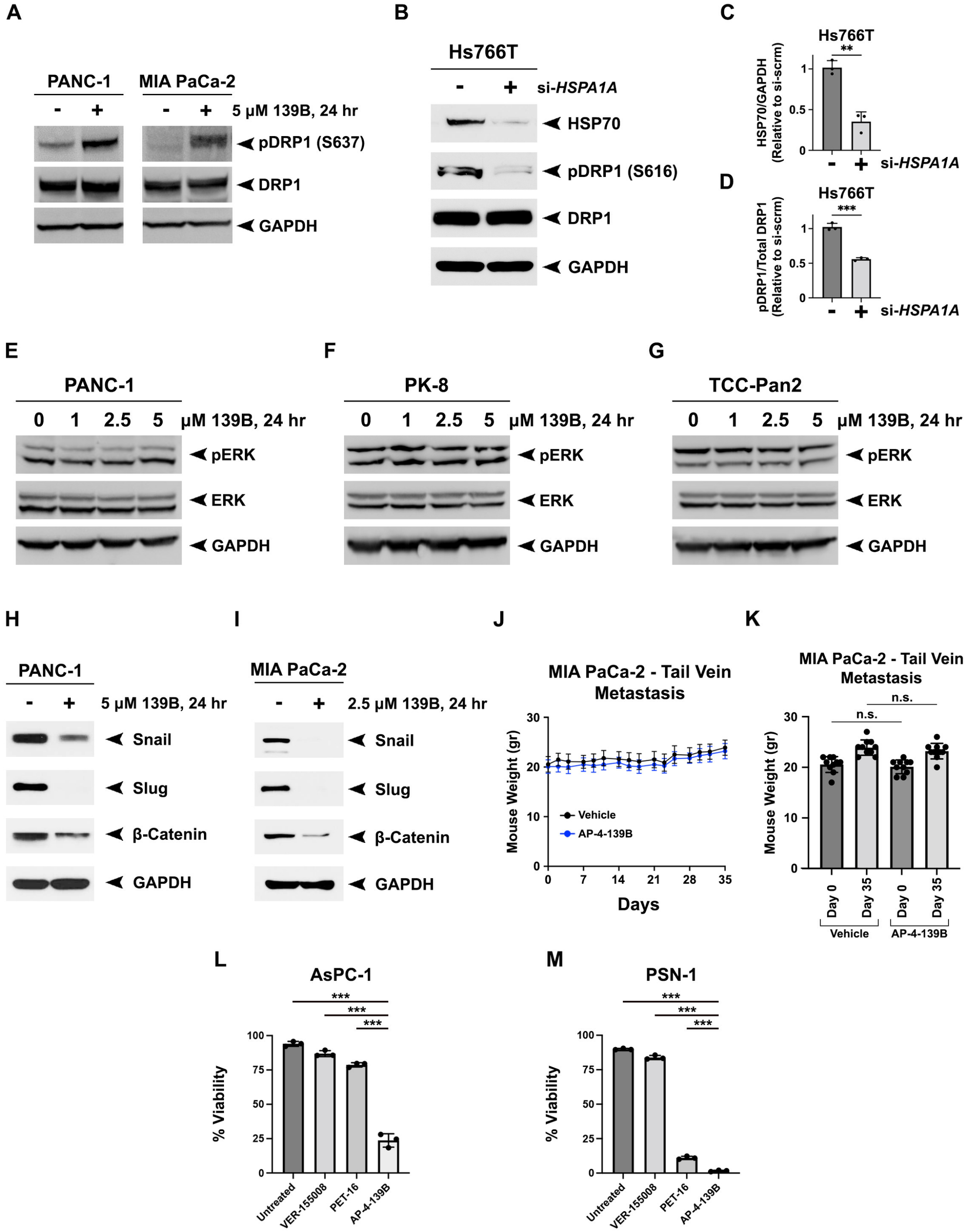

Supplemental Figure 3. HSP70 inhibition affects DRP1 phosphorylation in an ERK-independent manner and suppresses the metastatic potential of PDAC cells *in vitro* and *in vivo*.

A. PANC-1 and MIA PaCa-2 cells were treated with 5 μM AP-4-139B for 24 hours. Cell lysates were subjected to Western blot analysis and immunoblotted for phospho-DRP1 (S637), total DRP1, and GAPDH (loading control). n=2 independent experiments.

B. Hs766T cells were transfected with a pool of HSP70 siRNA and were harvested 48 hours later. Cell lysates were subjected to Western blot analysis and immunoblotted for phospho-DRP1 (S616), total DRP1, HSP70 and GAPDH (loading control).

C-D. Quantification of (B) was performed by obtaining the density of the phospho-DRP1 bands using ImageJ software and normalizing to the level of total DRP1. quantification of HSP70 was normalized to GAPDH. $**p<0.01$, $***p<0.001$. n=three independent experiments.

E-G. PANC-1, PK-8, and TCC-Pan2 cells were treated with the indicated doses of AP-4-139B for 24 hours. Cell lysates were subjected to Western blot analysis and immunoblotted for phospho-ERK, total ERK, and GAPDH (loading control). n=2 independent experiments.

H-I. PANC1 (H) and MIA PaCa-2 (I) cells were treated with DMSO (control) or 5μM AP-4-139B (or "139B") for 24 hours. Lysates were extracted and analyzed by Western blot analysis for protein levels of Snail, Slug, β-Catenin, and GAPDH (loading control). n=2 biological replicates.

J. Body weights of mice injected with MIA PaCa-2 cells for tail vein mediated lung metastasis. Weights were measured from the start of the treatment to determine mouse toxicity. n=8 mice per group.

K. Quantification of (J) at endpoint. Changes in total mouse weight between Day 0 and Day 35 in vehicle and AP-4-139B treatment groups are shown. Data is shown as the mouse weight ± the standard deviation; n.s., not significant.

L-M. AsPC-1 and PSN-1 cells were treated with 10 μM of VER-155008, PET-16, or AP-4-139B for 48 hours. Cells were then subjected to viability assays using trypan blue exclusion. $***p<0.001$. n=3 independent experiments.

Supplemental Figure 4

A

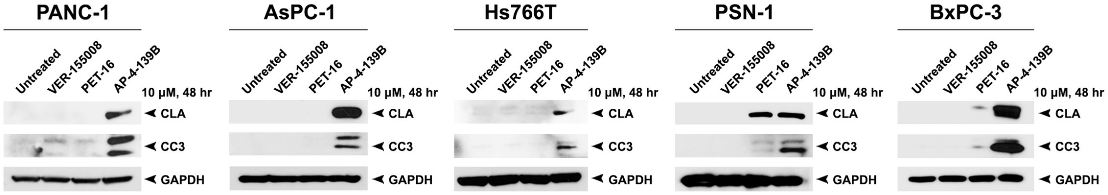

B

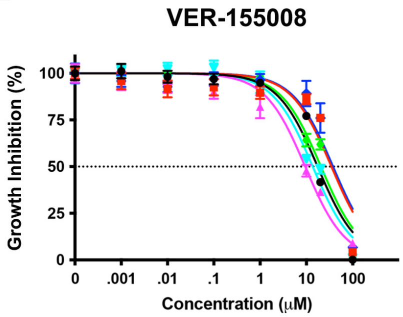

	MIA PaCa-2	PANC-1	AsPC-1	Hs766T	BxPC-3	PSN-1
GI50	17.49	34.79	9.422	37.59	13.72	20.60

C

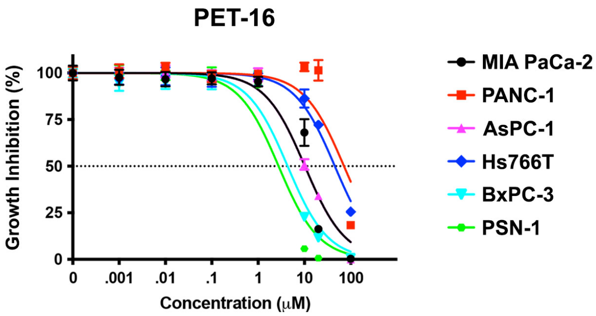

	MIA PaCa-2	PANC-1	AsPC-1	Hs766T	BxPC-3	PSN-1
GI50	10.23	71.49	10.22	45.33	4.341	2.772

D

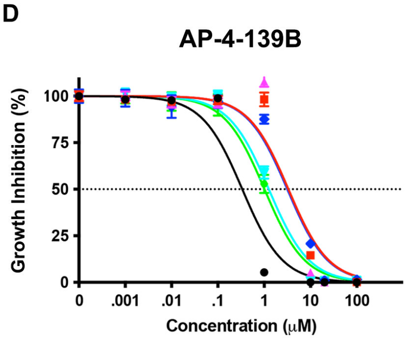

	MIA PaCa-2	PANC-1	AsPC-1	Hs766T	BxPC-3	PSN-1
GI50	0.3366	3.331	3.319	3.160	1.260	1.014

E

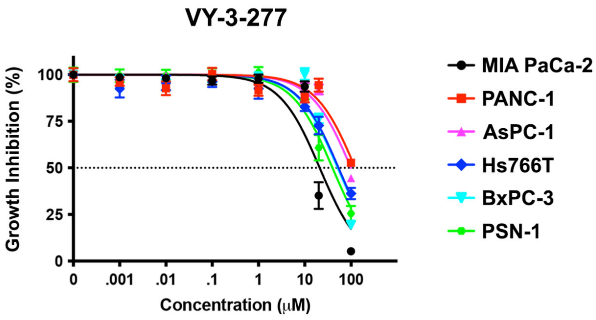

	MIA PaCa-2	PANC-1	AsPC-1	Hs766T	BxPC-3	PSN-1
GI50	21.02	123.6	99.93	53.61	51.27	39.69

F

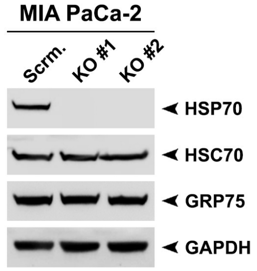

G

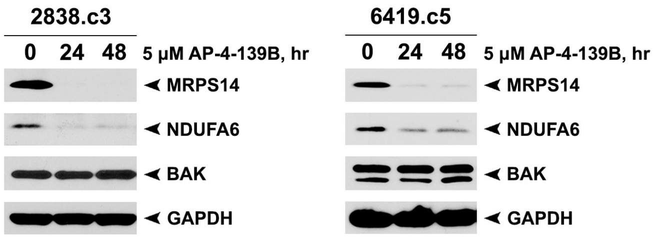

Supplemental Figure 4. AP-4-139B is a superior HSP70 inhibitor that leads to a reduction of HSP70 client proteins and induces cell death in PDAC cells.

A. Five human PDAC cell lines (PANC1, AsPC-1, Hs766T, PSN-1, and BxPC-3) were plates in 10 cm dishes at a concentration of $5x10^5$ cells per plate. The next day, cells were treated with 10 μM of the indicated inhibitors for 48 hours. Cell lysates were then subjected to Western blot analysis, and immunoblotted for Cleaved Lamin A (CLA), Cleaved Caspase 3 (CC3), and GAPDH (loading control).

B-E. A panel of six human PDAC cell lines (MIA PaCa-2, PANC1, AsPC-1, Hs766T, BxPC-3, and PSN-1) were treated with the indicated inhibitors for 72 hours and subjected to Alamar Blue assays. GI_{50} values are from six technical replicates and two biological replicates.

F. HSP70 knockout (KO) was confirmed by Western blot analysis. Cell lysates from two independent KO clones and a control (sg-scrambled) pool were subjected to Western blot analysis and immunoblotted for HSP70, HSC70, and GRP75, and GAPDH (loading control).

G. Mouse PDAC cell lines (2838c.3 and 6419c.5) were treated with 5μM of AP-4-139B and harvested at the indicated timepoints. Cell lysates were processed for Western blot analysis and immunoblotted for MRPS14, NDUFA6, BAK, and GAPDH (loading control).

Supplemental Figure 5

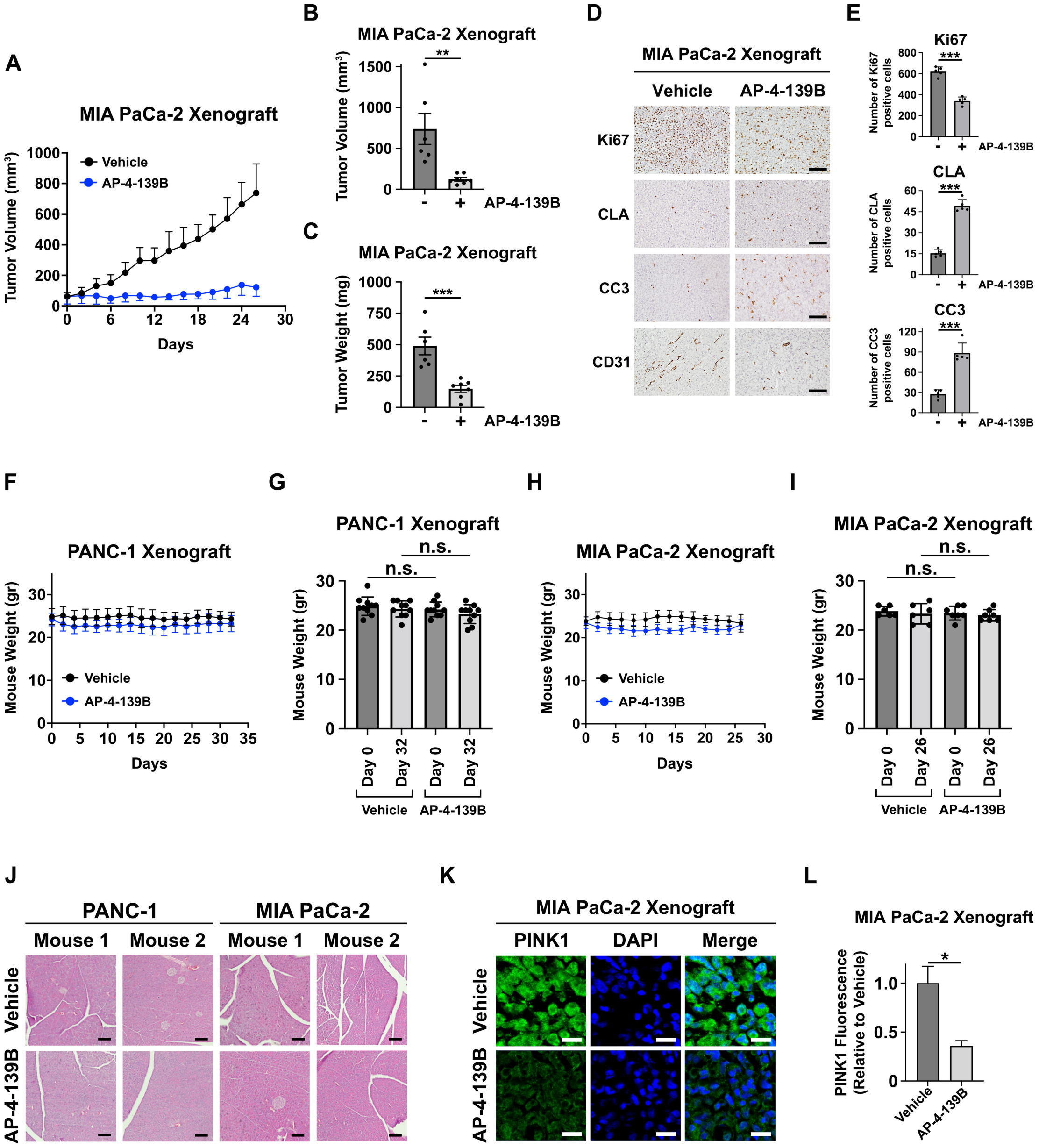

Supplemental Figure 5. HSP70 inhibition limits PDAC tumor progression *in vivo*.

A. $2.5x10^6$ MIA PaCa-2 cells were injected subcutaneously into the right flanks of 8–10-week-old female NSG mice. Once tumors reached an approximate size of 75 mm^3, mice were randomly sorted into vehicle and AP-4-139B treated groups. AP-4-139B was treated at a dose of 10mg/kg every other day, and tumors were measured every other day using a digital caliper. n=6-7 mice per group.

B-C. Quantification of tumor volume (B) and tumor weight (C) at endpoint; **p<0.01, ***p<0.001.

D. IHC analysis of MIA PaCa-2 xenograft tumors treated with AP-4-139B. Shown are representative images (five random fields of view per condition) of Ki67, Cleaved Lamin A, Cleaved Caspase 3, and CD31. n=5 mice per group; scale bar: 100 µm.

E. Quantification of (D); ***p<0.001.

F-I. Body weights of mice injected with PANC1 (F-G) and MIA PaCa-2 (H-I) tumor xenografts. Weights were measured at the start of the treatment to determine mouse toxicity. (G, I) Quantification of change in total mouse weight from F and H. n.s. not significant, n=6-10 mice per group.

J. H&E staining of pancreas tissues of mice injected with PANC1 or MIA PaCa-2 xenografts. Tissues were collected at the end of the study. Bar scale: 100µm.

K. Immunofluorescence (IF) analysis of PINK1 (green) was performed on MIA PaCa-2 xenograft tumors, and counterstained with DAPI (blue). Bar scale: 20 µm.

L. Quantification of (K); n=3 mice per group. *p<0.05.

Supplemental Figure 6

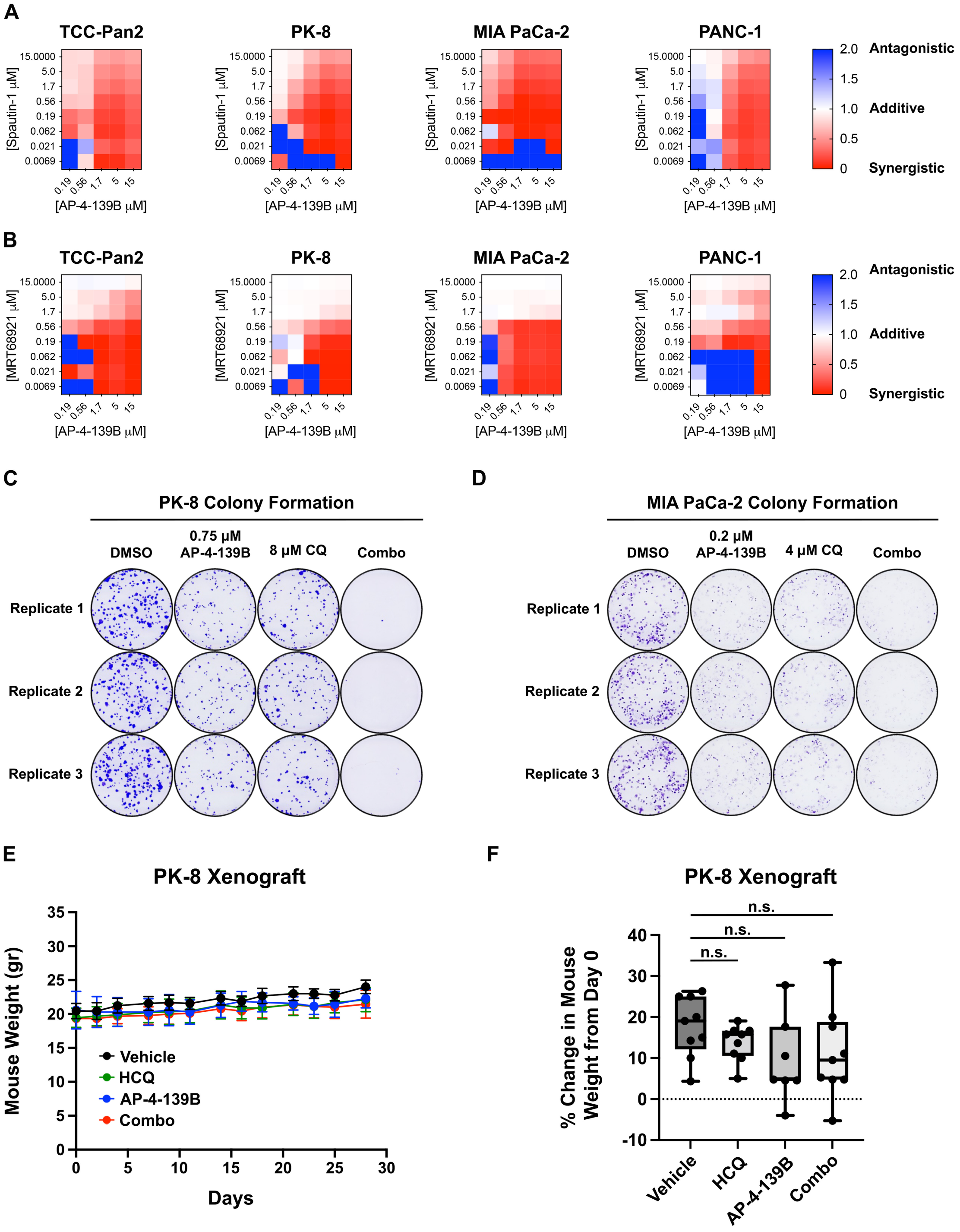

476

477

Supplemental Figure 6. AP-4-139B synergizes with autophagy inhibition in human PDAC cell lines and in a xenograft model.

A-B. TCC-Pan2, PK-8, MIA PaCa-2, and PANC-1 cells were treated for 72 hours with HSP70i (AP-4-139) in combination with one of the following autophagy inhibitors, Spautin-1 or MRT68921, at the indicated concentrations. Cells were stained for viability with Calcein and imaged using a Celigo image cytometer. Cell numbers at endpoint were normalized to vehicle-treated control (100% growth) for each cell line. Heatmaps representing BLISS independence scores corresponding using proliferation indices for each condition. Scores <1 indicate synergy (red), score =1 indicated additivity (white), and scores >1 indicate antagonism (blue). n=3 independent experiments.

C-D. PK-8 and MIA PaCa-2 cells were seeded in 60-mm dishes at a concentration of 500 cells per dish and were subjected to colony formation assays in the presence of the indicated compounds: AP-4-139B, CQ, or the combination of both compounds (Combo). Cells were fixed and stained with 0.5% Crystal Violet after seven (MIA PaCa-2) or fourteen (PK-8) days. Shown are images of all three replicates of a single experiment; one replicate for each condition per cell line shown was used to generate Figure 7C-D. All colony formation assays were repeated three times.

E. Relative mouse weights throughout the course of the PK-8 xenograft mouse study described in Figure 7E-F. Mouse weights were measured three times per week for the entire 28-day course of treatment. n=7-9 mice per group.

F. Quantification of percent change in PK-8 xenograft mouse weights assessed over 28 days treated with the following: 1. Vehicle (control), 2. Hydroxychloroquine (50 mg/kg daily), 3. AP-4-139B (10 mg/kg every other day), or 4. the combination of both agents at the doses indicated. n.s. not significant.

Supplementary References

1 Barnoud, T., Parris, J. L. D. & Murphy, M. E. Tumor cells containing the African-Centric S47 variant of TP53 show increased Warburg metabolism. *Oncotarget* **10**, 1217-1223, doi:10.18632/oncotarget.26660 (2019).

2 Basu, S., Barnoud, T., Kung, C. P., Reiss, M. & Murphy, M. E. The African-specific S47 polymorphism of p53 alters chemosensitivity. *Cell Cycle* **15**, 2557-2560, doi:10.1080/15384101.2016.1215390 (2016).

3 Barnoud, T., Donninger, H. & Clark, G. J. Ras Regulates Rb via NORE1A. *J Biol Chem* **291**, 3114-3123, doi:10.1074/jbc.M115.697557 (2016).

4 Donninger, H., Barnoud, T., Nelson, N., Kassler, S., Clark, J., Cummins, T. D. *et al.* RASSF1A and the rs2073498 Cancer Associated SNP. *Front Oncol* **1**, 54, doi:10.3389/fonc.2011.00054 (2011).

5 Barnoud, T., Budina-Kolomets, A., Basu, S., Leu, J. I., Good, M., Kung, C. P. *et al.* Tailoring Chemotherapy for the African-Centric S47 Variant of TP53. *Cancer Res* **78**, 5694-5705, doi:10.1158/0008-5472.CAN-18-1327 (2018).

6 Parris, J. L. D., Barnoud, T., Leu, J. I., Leung, J. C., Ma, W., Kirven, N. A. *et al.* HSP70 inhibition blocks adaptive resistance and synergizes with MEK inhibition for the treatment of NRAS-mutant melanoma. *Cancer Res Commun* **1**, 17-29, doi:10.1158/2767-9764.crc-21-0033 (2021).

7 Diehl, J. N., Klomp, J. E., Snare, K. R., Hibshman, P. S., Blake, D. R., Kaiser, Z. D. *et al.* The KRAS-regulated kinome identifies WEE1 and ERK coinhibition as a potential therapeutic strategy in KRAS-mutant pancreatic cancer. *J Biol Chem* **297**, 101335, doi:10.1016/j.jbc.2021.101335 (2021).

8 Foucquier, J. & Guedj, M. Analysis of drug combinations: current methodological landscape. *Pharmacol Res Perspect* **3**, e00149, doi:10.1002/prp2.149 (2015).

9 Bryant, K. L., Stalnecker, C. A., Zeitouni, D., Klomp, J. E., Peng, S., Tikunov, A. P. *et al.* Combination of ERK and autophagy inhibition as a treatment approach for pancreatic cancer. *Nat Med* **25**, 628-640, doi:10.1038/s41591-019-0368-8 (2019).

10 Hobbs, G. A., Baker, N. M., Miermont, A. M., Thurman, R. D., Pierobon, M., Tran, T. H. *et al.* Atypical KRAS(G12R) Mutant Is Impaired in PI3K Signaling and Macropinocytosis in Pancreatic Cancer. *Cancer Discov* **10**, 104-123, doi:10.1158/2159-8290.CD-19-1006 (2020).

11 Basu, S., Gnanapradeepan, K., Barnoud, T., Kung, C. P., Tavecchio, M., Scott, J. *et al.* Mutant p53 controls tumor metabolism and metastasis by regulating PGC-1alpha. *Genes Dev* **32**, 230-243, doi:10.1101/gad.309062.117 (2018).

12 Gnanapradeepan, K., Leu, J. I., Basu, S., Barnoud, T., Good, M., Lee, J. V. *et al.* Increased mTOR activity and metabolic efficiency in mouse and human cells containing the African-centric tumor-predisposing p53 variant Pro47Ser. *Elife* **9**, doi:10.7554/eLife.55994 (2020).

13 Bertolini, I., Ghosh, J. C., Kossenkov, A. V., Mulugu, S., Krishn, S. R., Vaira, V. *et al.* Small Extracellular Vesicle Regulation of Mitochondrial Dynamics Reprograms a Hypoxic Tumor Microenvironment. *Dev Cell* **55**, 163-177 e166, doi:10.1016/j.devcel.2020.07.014 (2020).

14 Bertolini, I., Keeney, F. & Altieri, D. C. Protocol for assessing real-time changes in mitochondrial morphology, fission and fusion events in live cells using confocal microscopy. *STAR Protoc* **2**, 100767, doi:10.1016/j.xpro.2021.100767 (2021).

15 Budina-Kolomets, A., Webster, M. R., Leu, J. I., Jennis, M., Krepler, C., Guerrini, A. *et al.* HSP70 Inhibition Limits FAK-Dependent Invasion and Enhances the Response to Melanoma Treatment with BRAF Inhibitors. *Cancer Res* **76**, 2720-2730, doi:10.1158/0008-5472.CAN-15-2137 (2016).

16 Bertolini, I., Perego, M., Ghosh, J. C., Kossenkov, A. V. & Altieri, D. C. NFkappaB activation by hypoxic small extracellular vesicles drives oncogenic reprogramming in a breast cancer microenvironment. *Oncogene* **41**, 2520-2525, doi:10.1038/s41388-022-02280-3 (2022).

17 Akbulut, O., Lengerli, D., Saatci, O., Duman, E., Seker, U. O. S., Isik, A. *et al.* A Highly Potent TACC3 Inhibitor as a Novel Anticancer Drug Candidate. *Mol Cancer Ther* **19**, 1243-1254, doi:10.1158/1535-7163.MCT-19-0957 (2020).

18 Assidicky, R., Tokat, U. M., Tarman, I. O., Saatci, O., Ersan, P. G., Raza, U. *et al.* Targeting HIF1-alpha/miR-326/ITGA5 axis potentiates chemotherapy response in triple-negative breast cancer. *Breast Cancer Res Treat* **193**, 331-348, doi:10.1007/s10549-022-06569-5 (2022).

19 Saatci, O., Kaymak, A., Raza, U., Ersan, P. G., Akbulut, O., Banister, C. E. *et al.* Targeting lysyl oxidase (LOX) overcomes chemotherapy resistance in triple negative breast cancer. *Nat Commun* **11**, 2416, doi:10.1038/s41467-020-16199-4 (2020).

20 Goldman, M. J., Craft, B., Hastie, M., Repecka, K., McDade, F., Kamath, A. *et al.* Visualizing and interpreting cancer genomics data via the Xena platform. *Nat Biotechnol* **38**, 675-678, doi:10.1038/s41587-020-0546-8 (2020).

21 Moffitt, R. A., Marayati, R., Flate, E. L., Volmar, K. E., Loeza, S. G., Hoadley, K. A. *et al.* Virtual microdissection identifies distinct tumor- and stroma-specific subtypes of pancreatic ductal adenocarcinoma. *Nat Genet* **47**, 1168-1178, doi:10.1038/ng.3398 (2015).

22 Barnoud, T., Leung, J. C., Leu, J. I., Basu, S., Poli, A. N. R., Parris, J. L. D. *et al.* A Novel Inhibitor of HSP70 Induces Mitochondrial Toxicity and Immune Cell Recruitment in Tumors. *Cancer Res* **80**, 5270-5281, doi:10.1158/0008-5472.CAN-20-0397 (2020).
