# Supplementary material for: HSP70-mediated mitochondrial dynamics and autophagy represent a novel vulnerability in pancreatic cancer
Source: Cell Death Differ. 2024 May 28;31(7):881–96. doi: 10.1038/s41418-024-01310-9 (PMC11239841; doi:10.1038/s41418-024-01310-9)

Figure 3

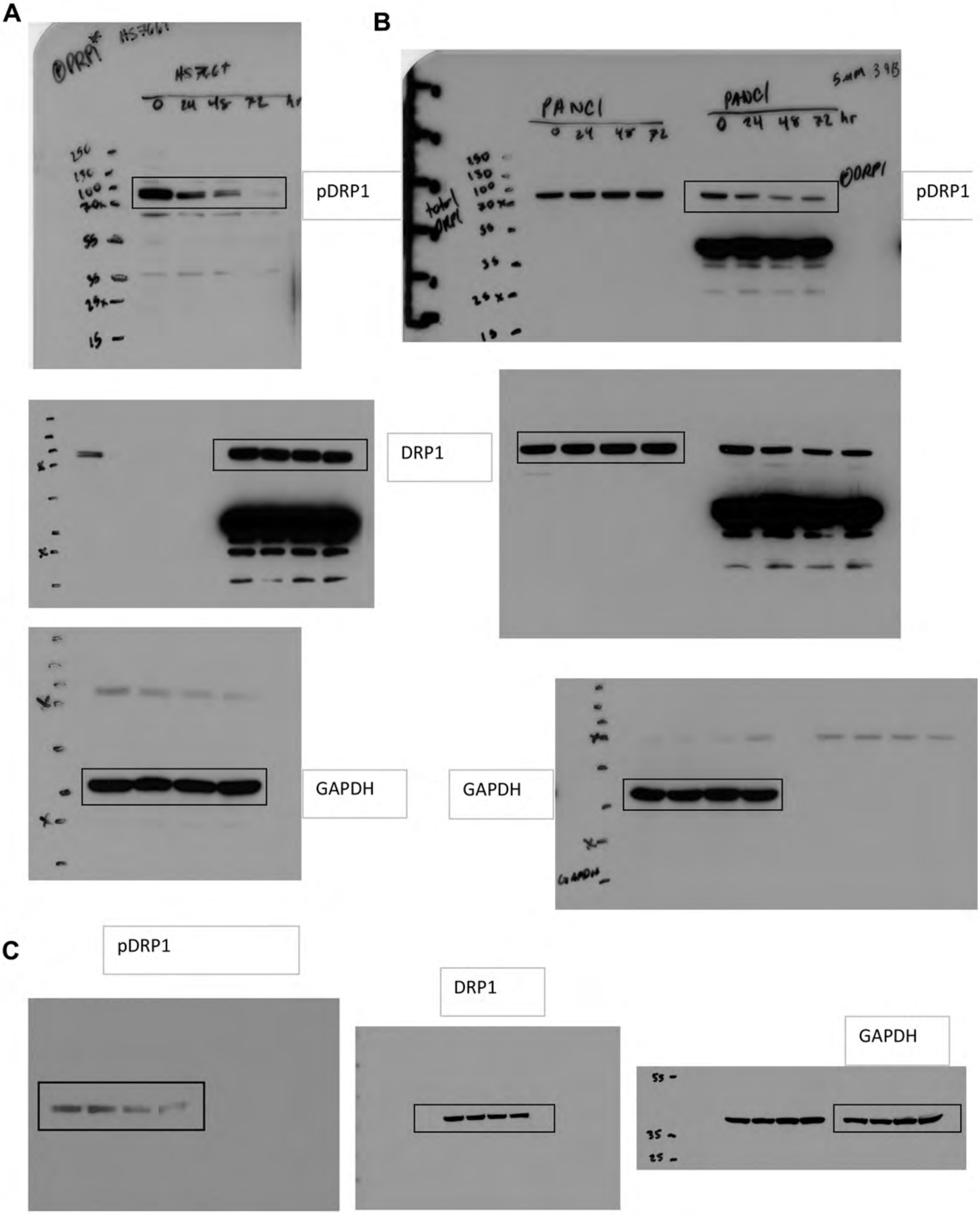
A
B
pDRP1
PANC1
0 24 48 72
0 24 48 72 hr
pDRP1
DRP1
GAPDH
GAPDH
C
pDRP1
DRP1
GAPDH
55
35
25

Figure 3

G

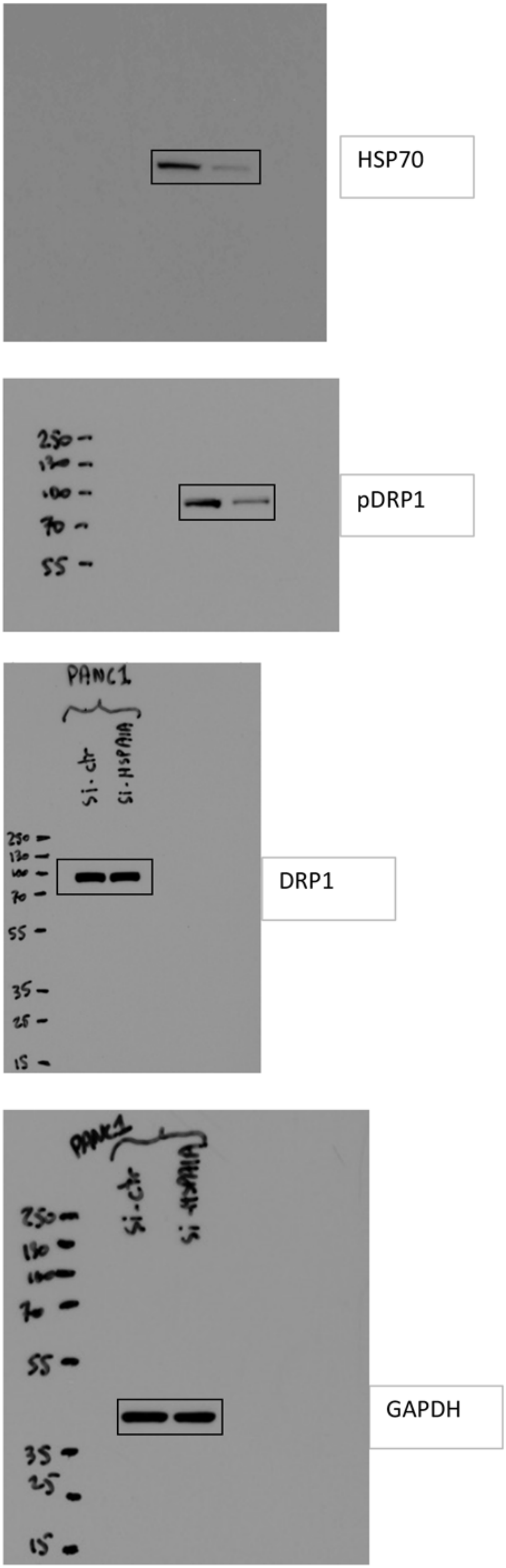

Figure 3

J (1-Hs766T)

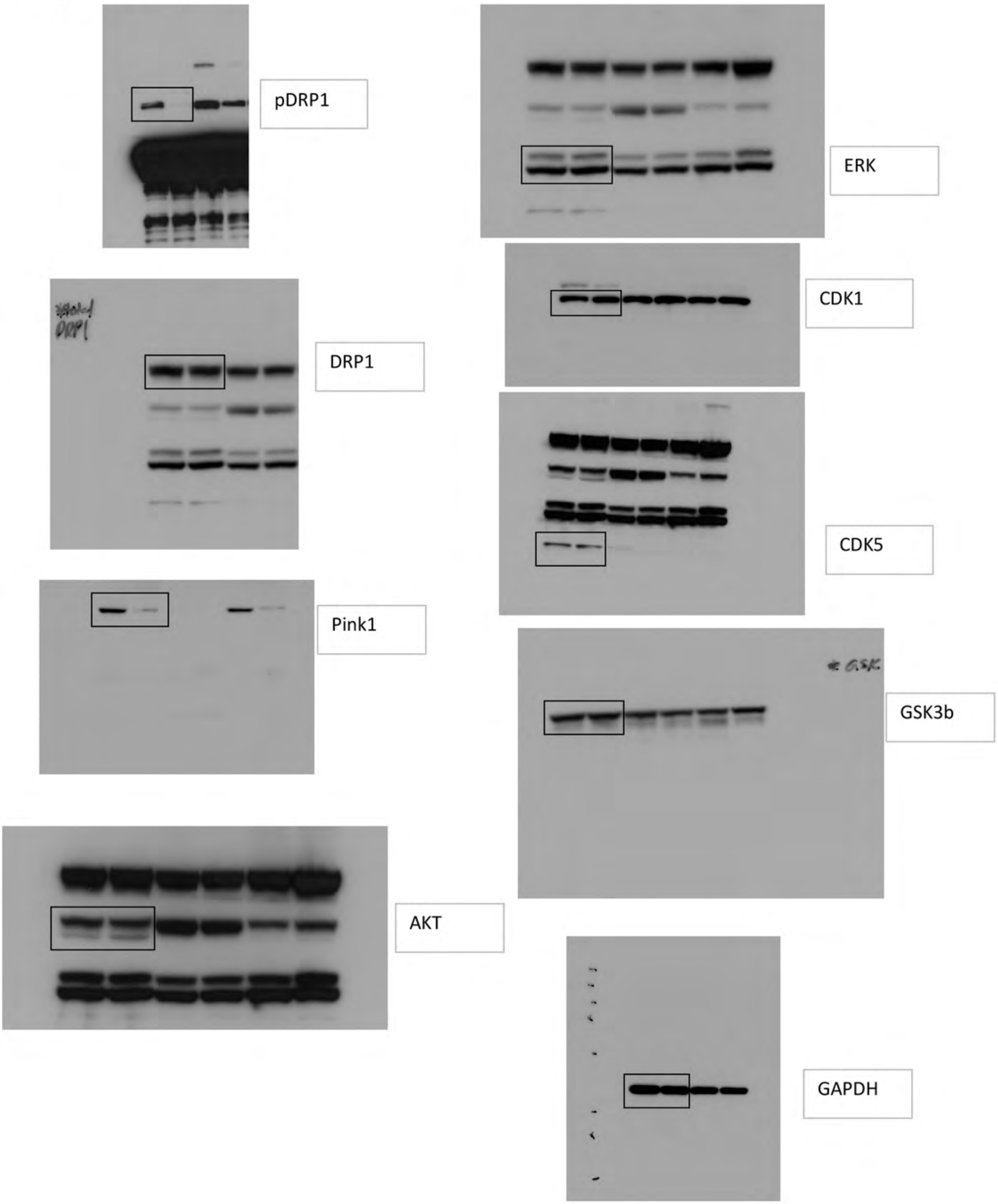

Figure 3

J (2)

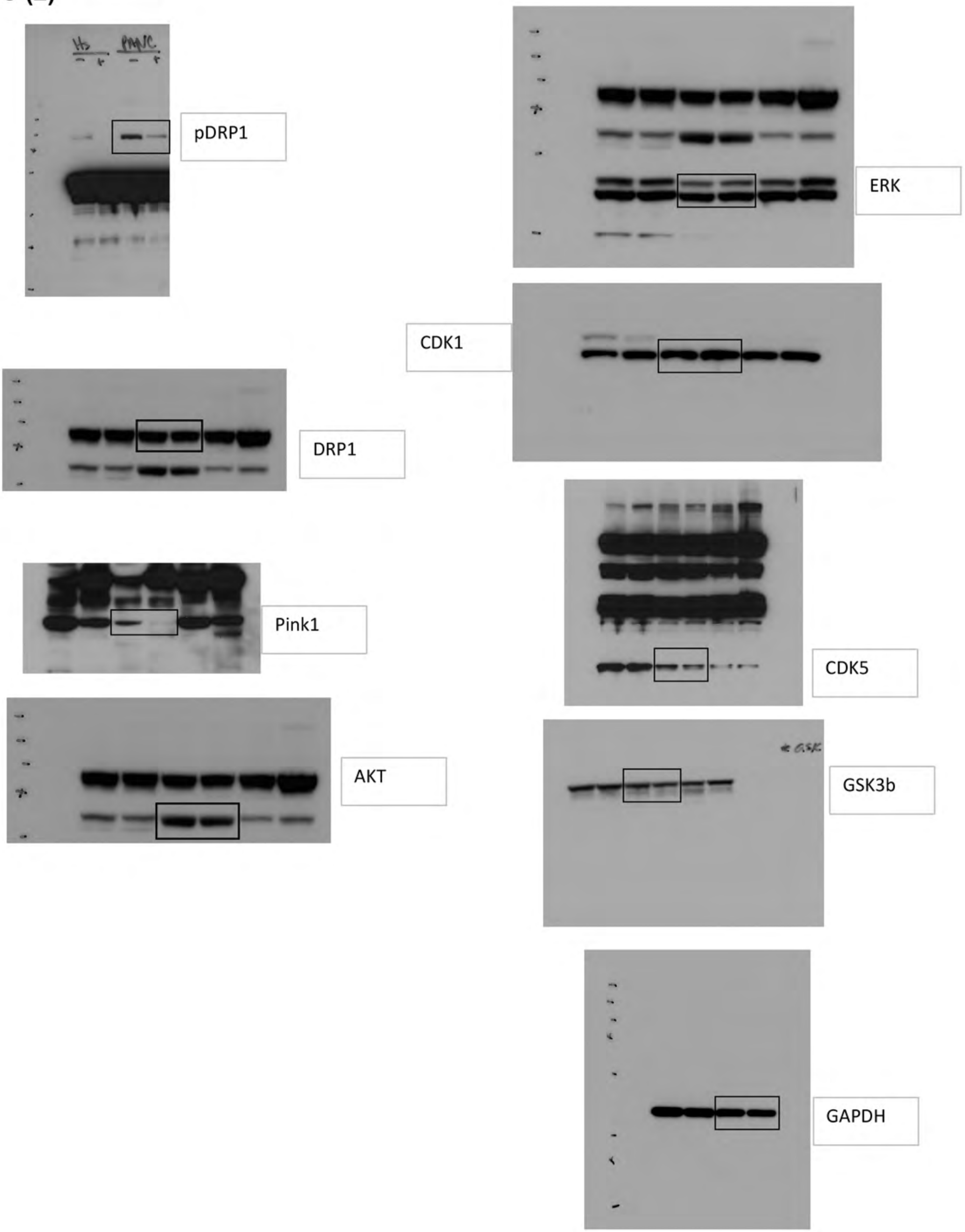

Figure 3

L

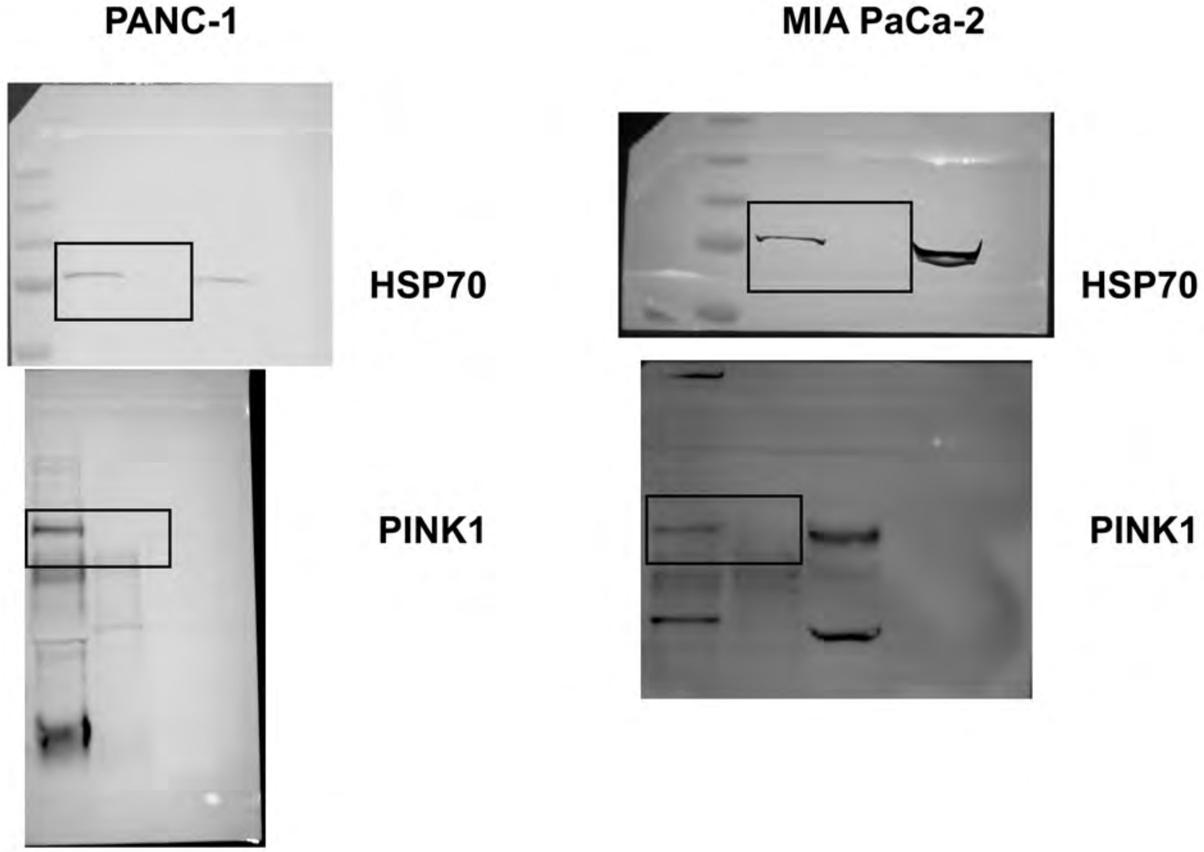
PANC-1
MIA PaCa-2
HSP70
HSP70
PINK1
PINK1

N

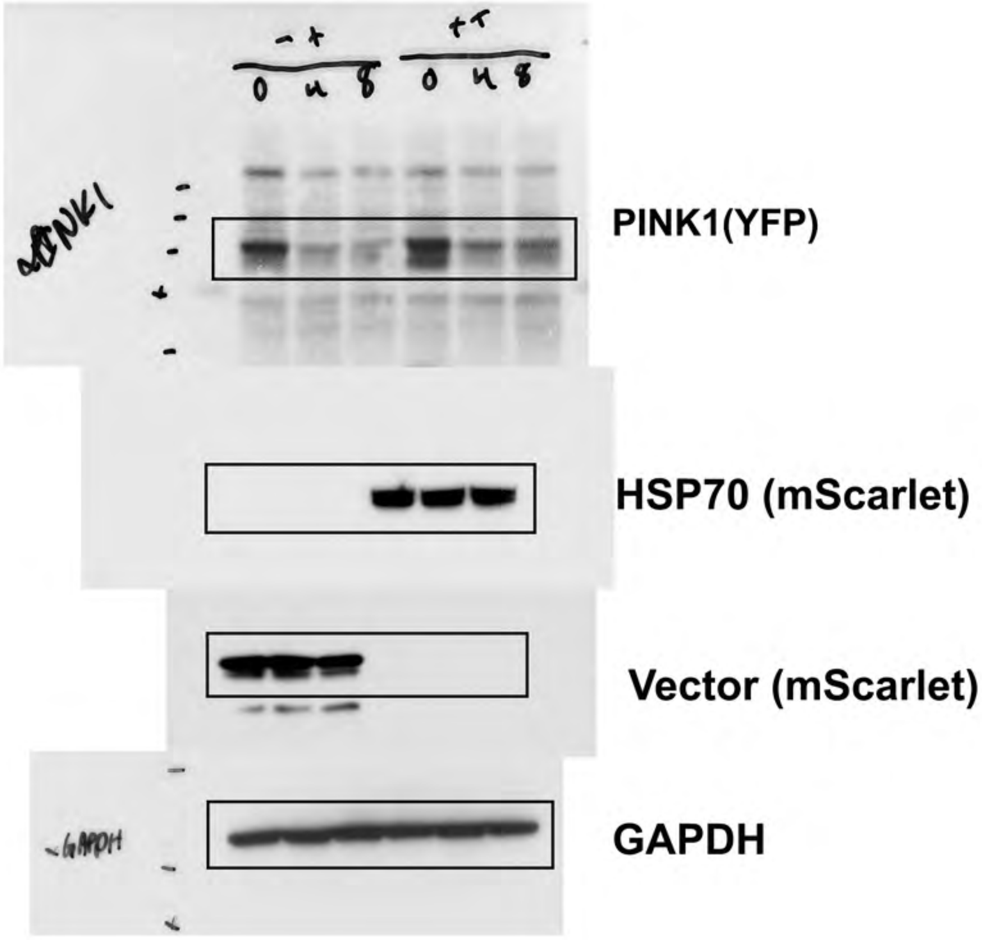
PINK1(YFP)
HSP70 (mScarlet)
Vector (mScarlet)
GAPDH

Figure 5

E

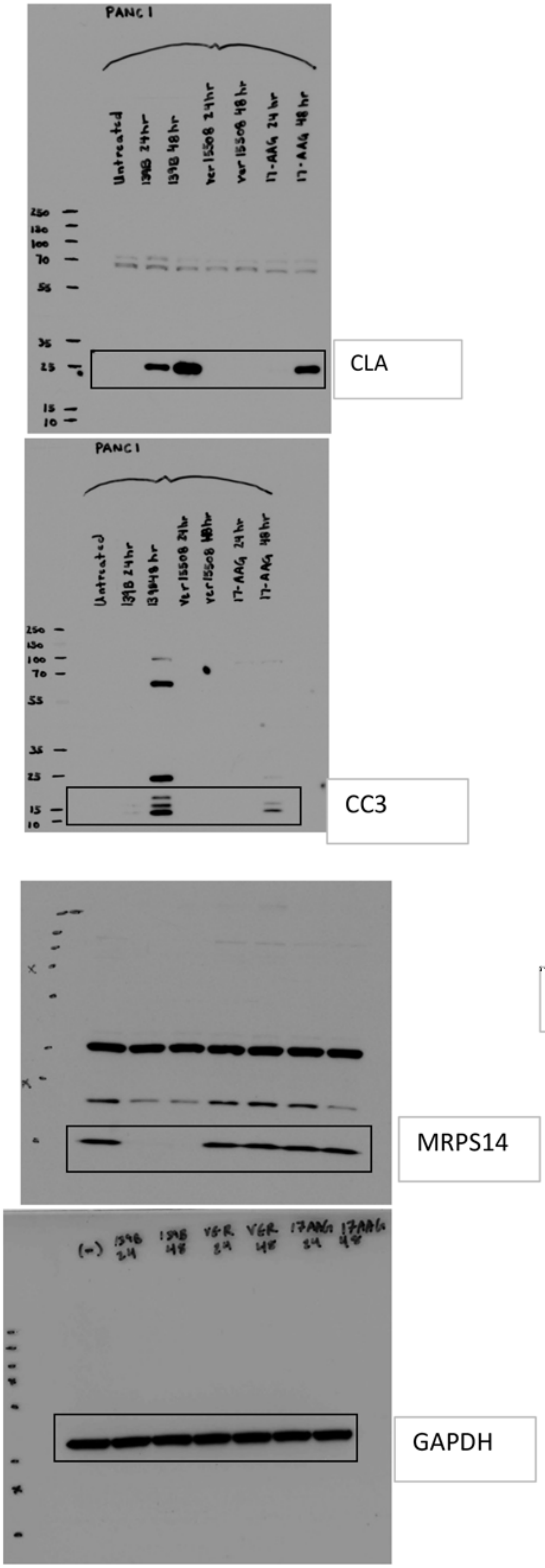

G (1)-PANC-1

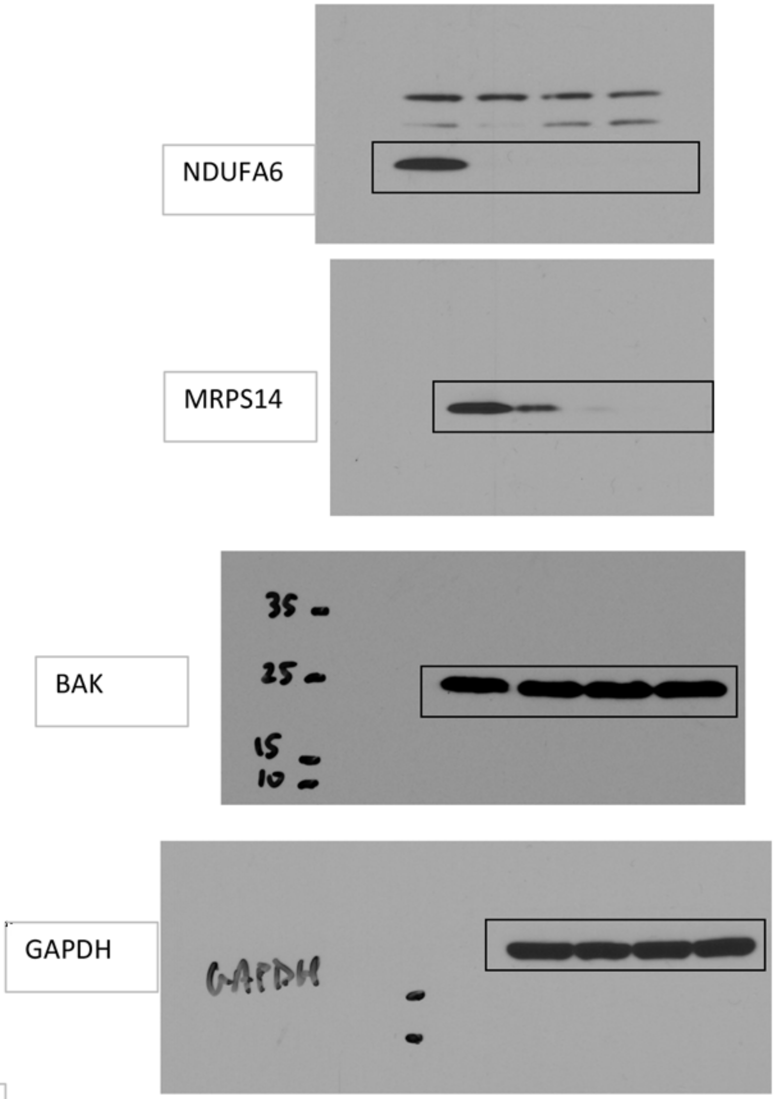

Figure 5

G (2)- MIA PaCa-2

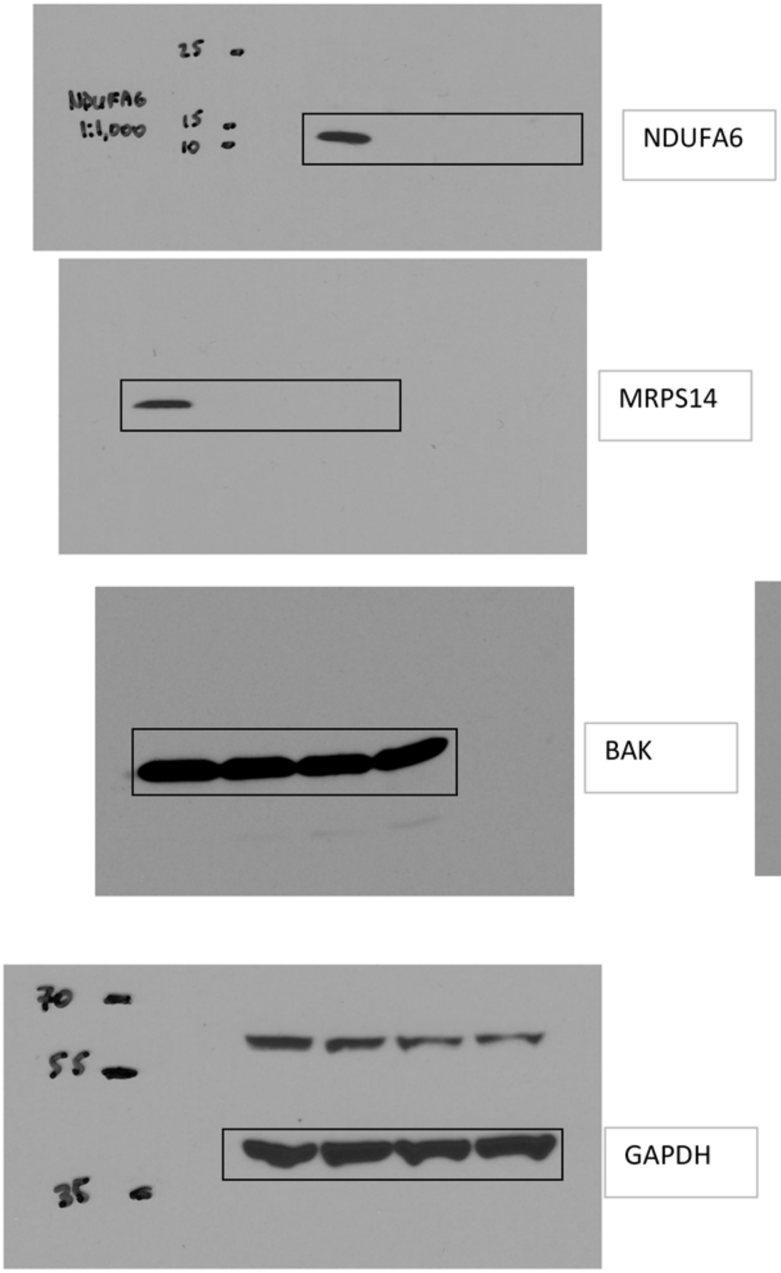

G (3)- Hs766T

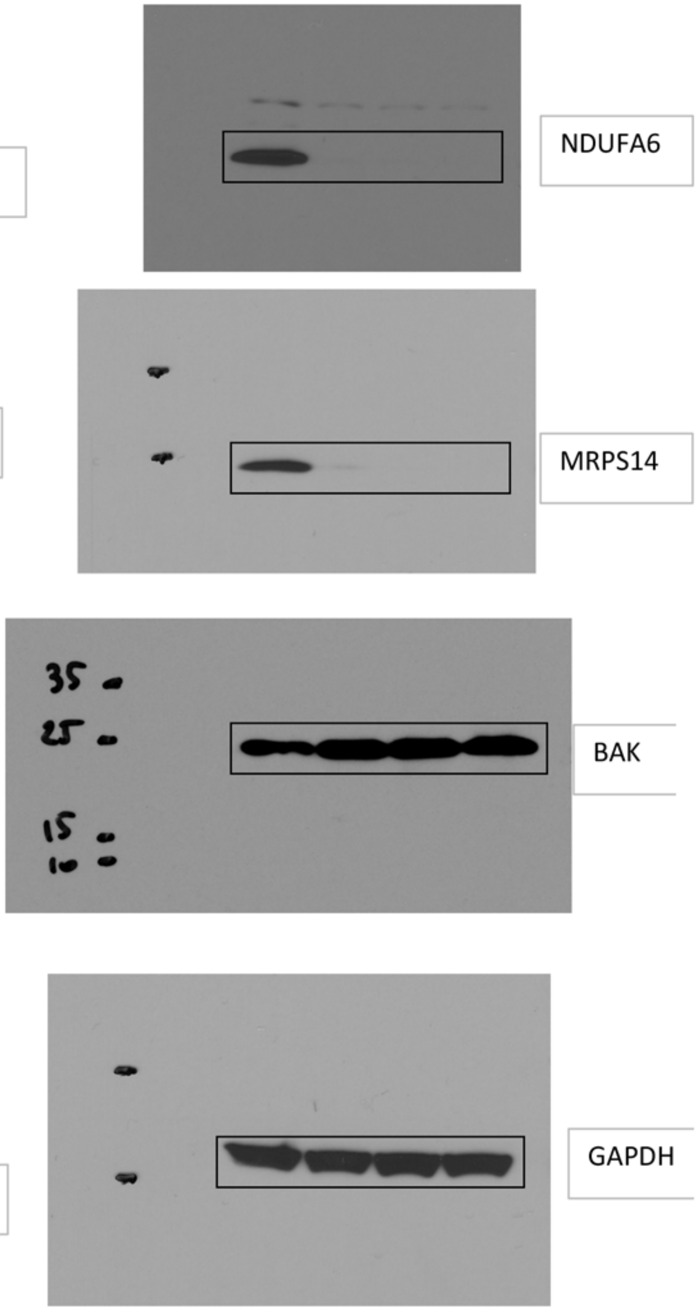

Figure 6

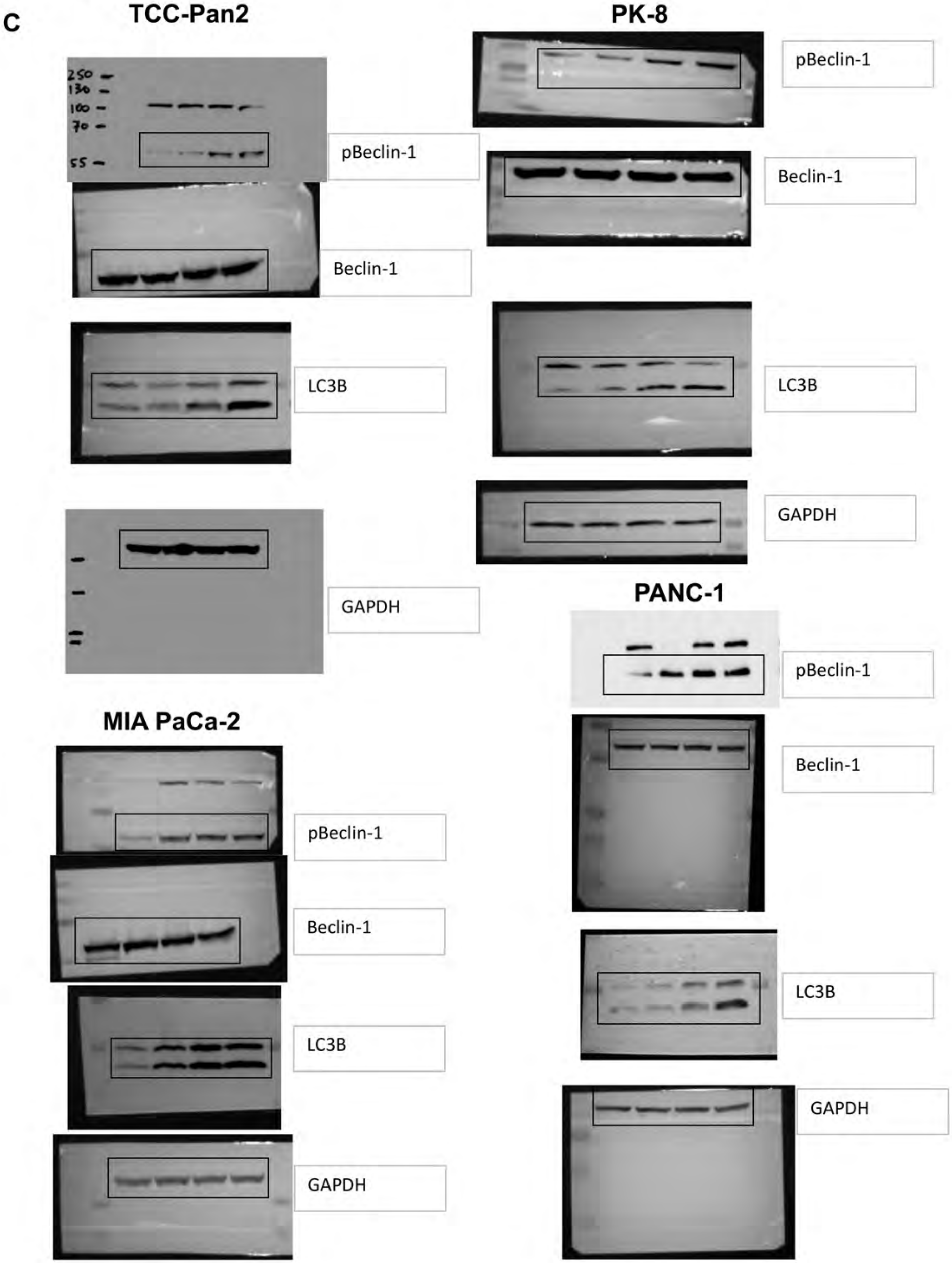
C
TCC-Pan2
250
130
100
70
55
pBeclin-1
Beclin-1
LC3B
GAPDH
PK-8
pBeclin-1
Beclin-1
LC3B
GAPDH
PANC-1
pBeclin-1
Beclin-1
LC3B
GAPDH
MIA PaCa-2
pBeclin-1
Beclin-1
LC3B
GAPDH

Figure 6

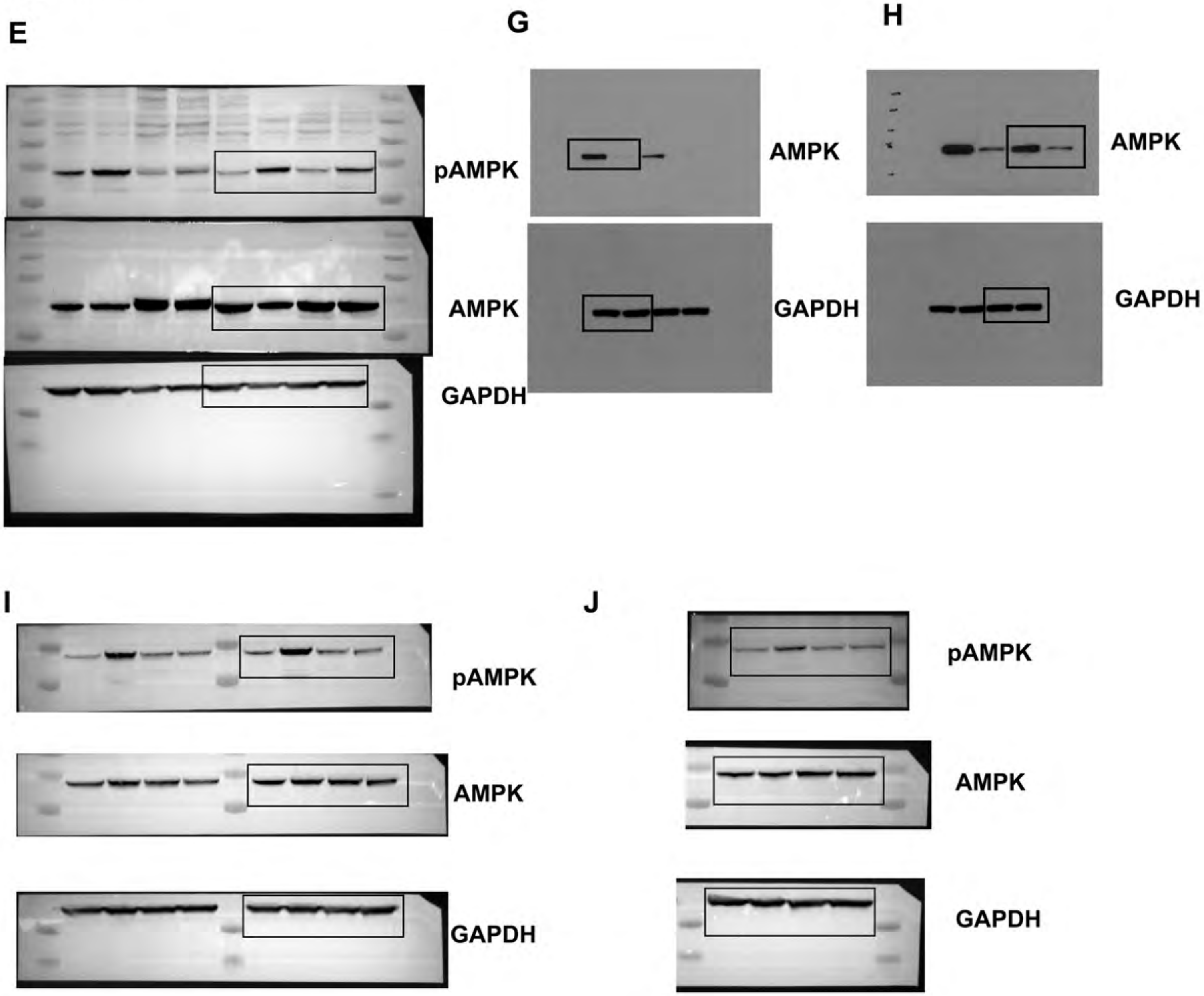
E
pAMPK
AMPK
GAPDH
G
AMPK
GAPDH
H
AMPK
GAPDH
I
pAMPK
AMPK
GAPDH
J
pAMPK
AMPK
GAPDH

Figure 7

B

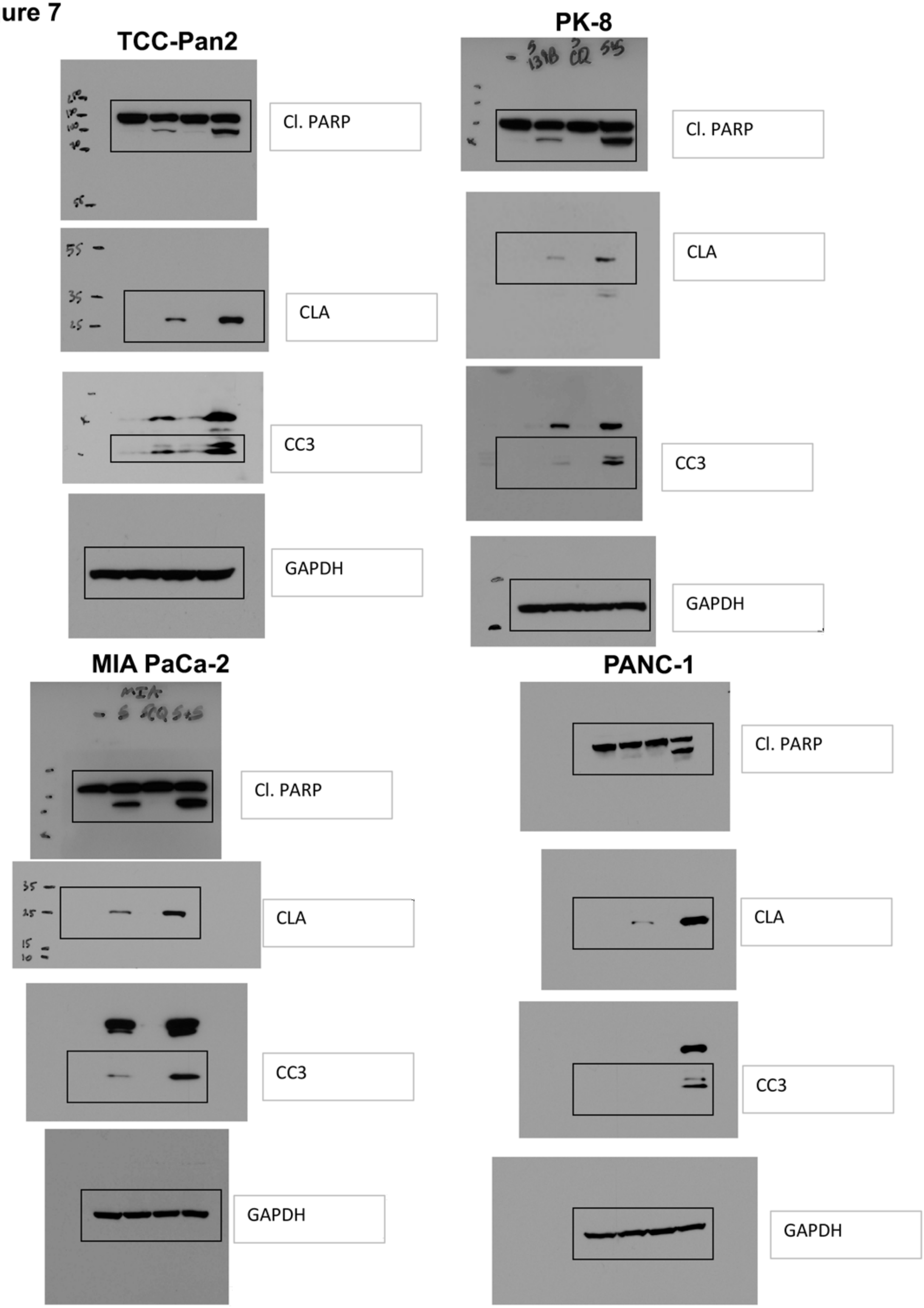
TCC-Pan2
PK-8
MIA PaCa-2
PANC-1
Cl. PARP
CLA
CC3
GAPDH
Cl. PARP
CLA
CC3
GAPDH
Cl. PARP
CLA
CC3
GAPDH
Cl. PARP
CLA
CC3
GAPDH

Supplemental Figure 1

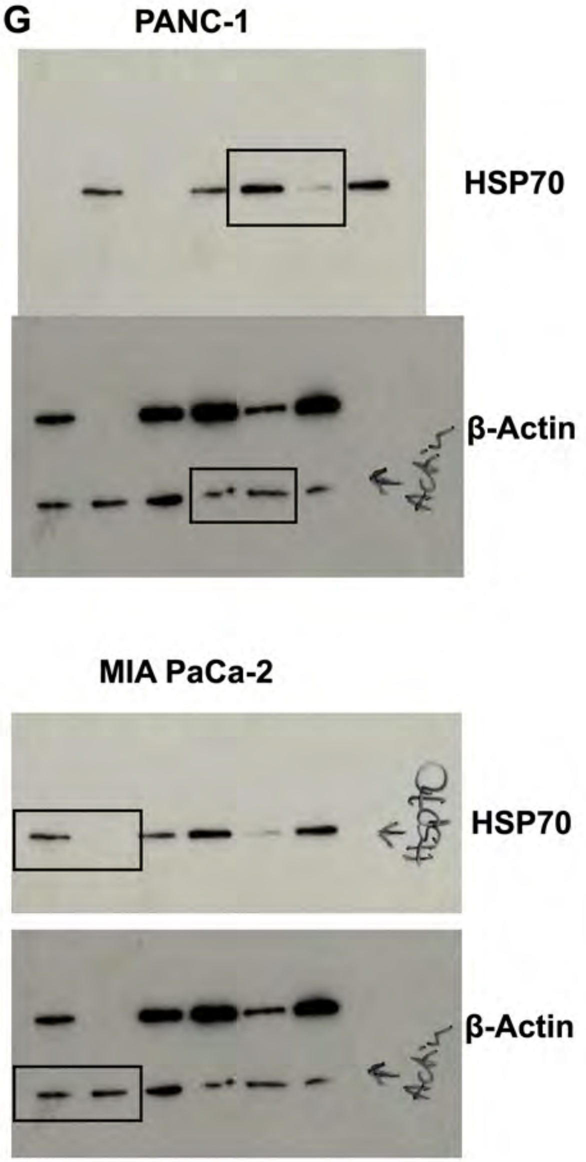

Supplemental Figure 3

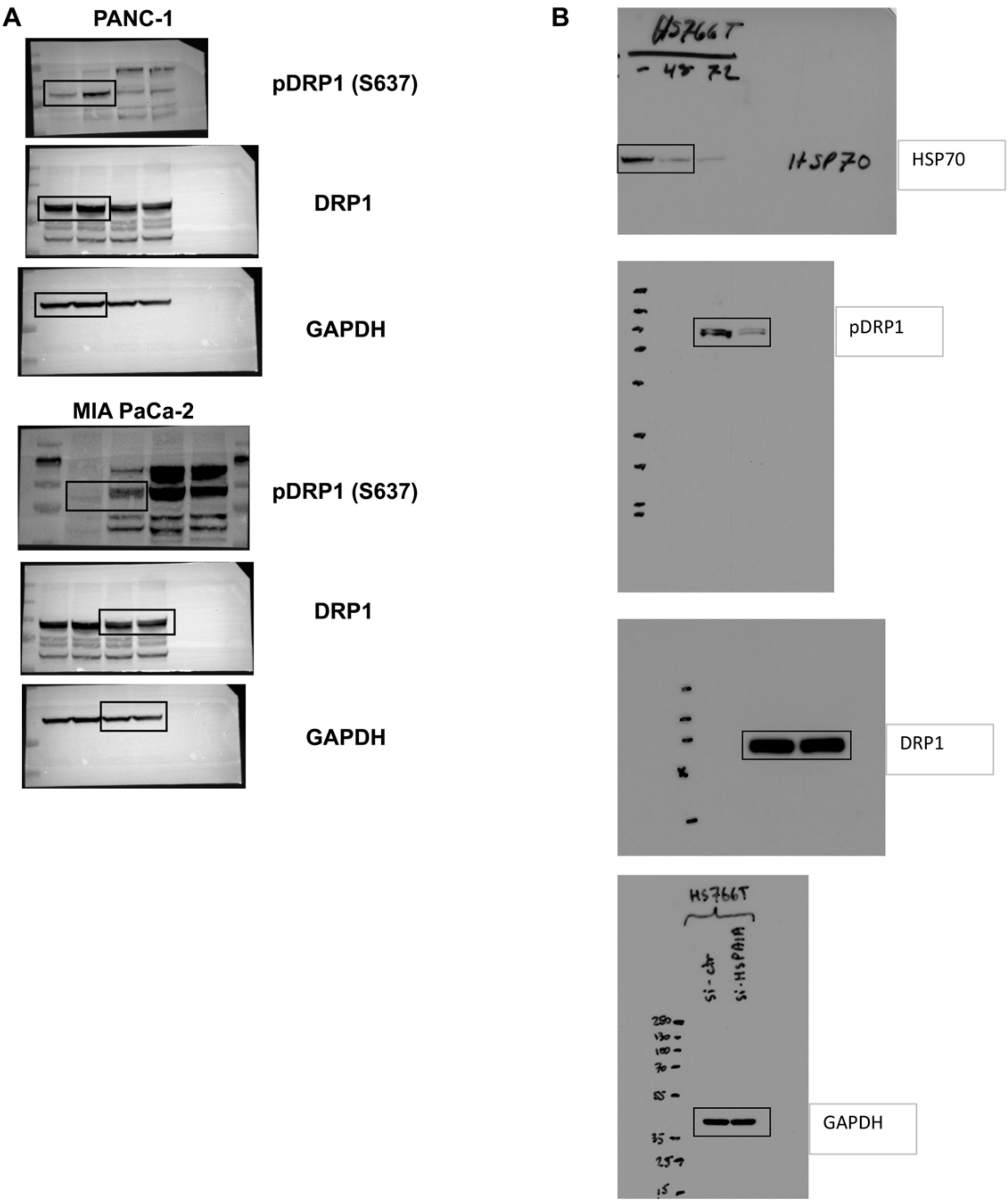

Supplemental Figure 3

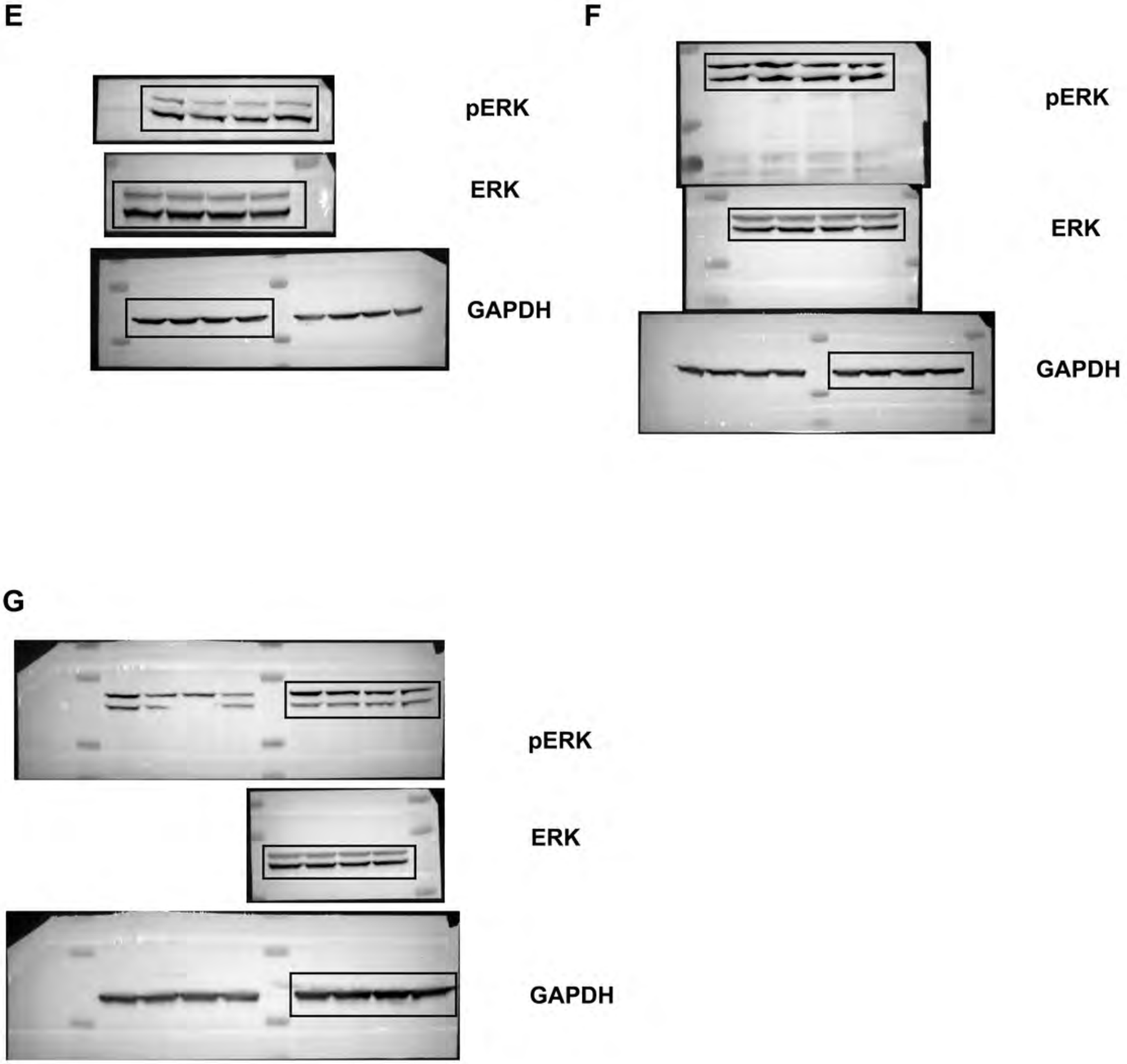

Supplemental Figure 3

H

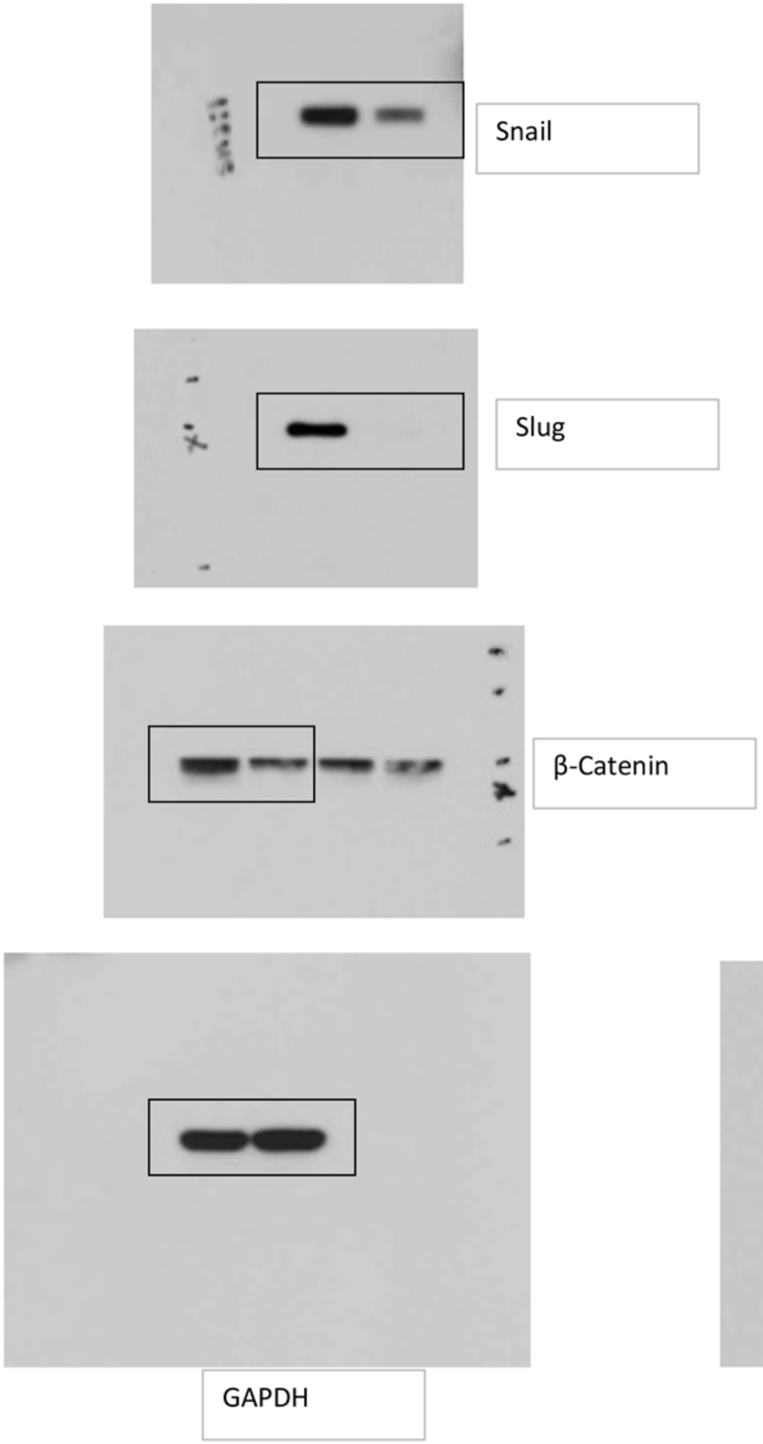

I

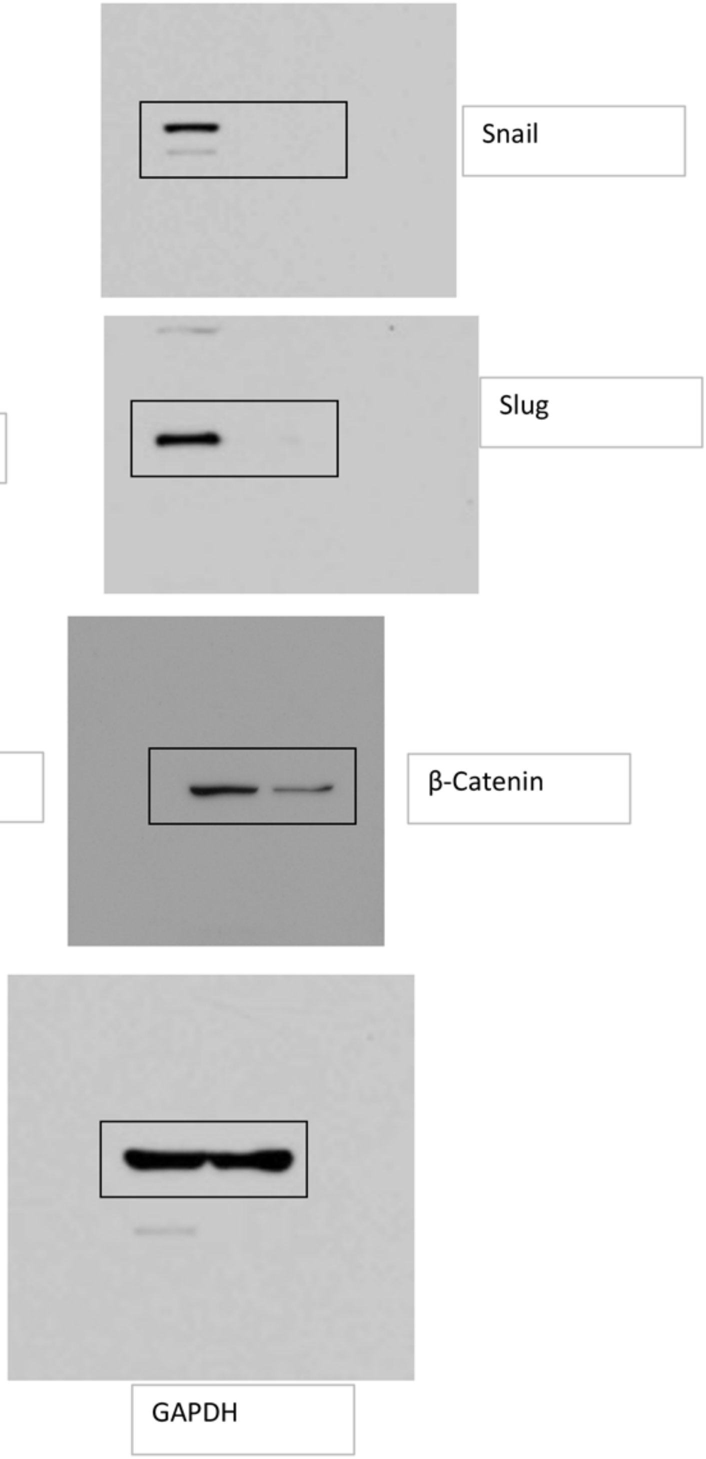

Supplemental Figure 4

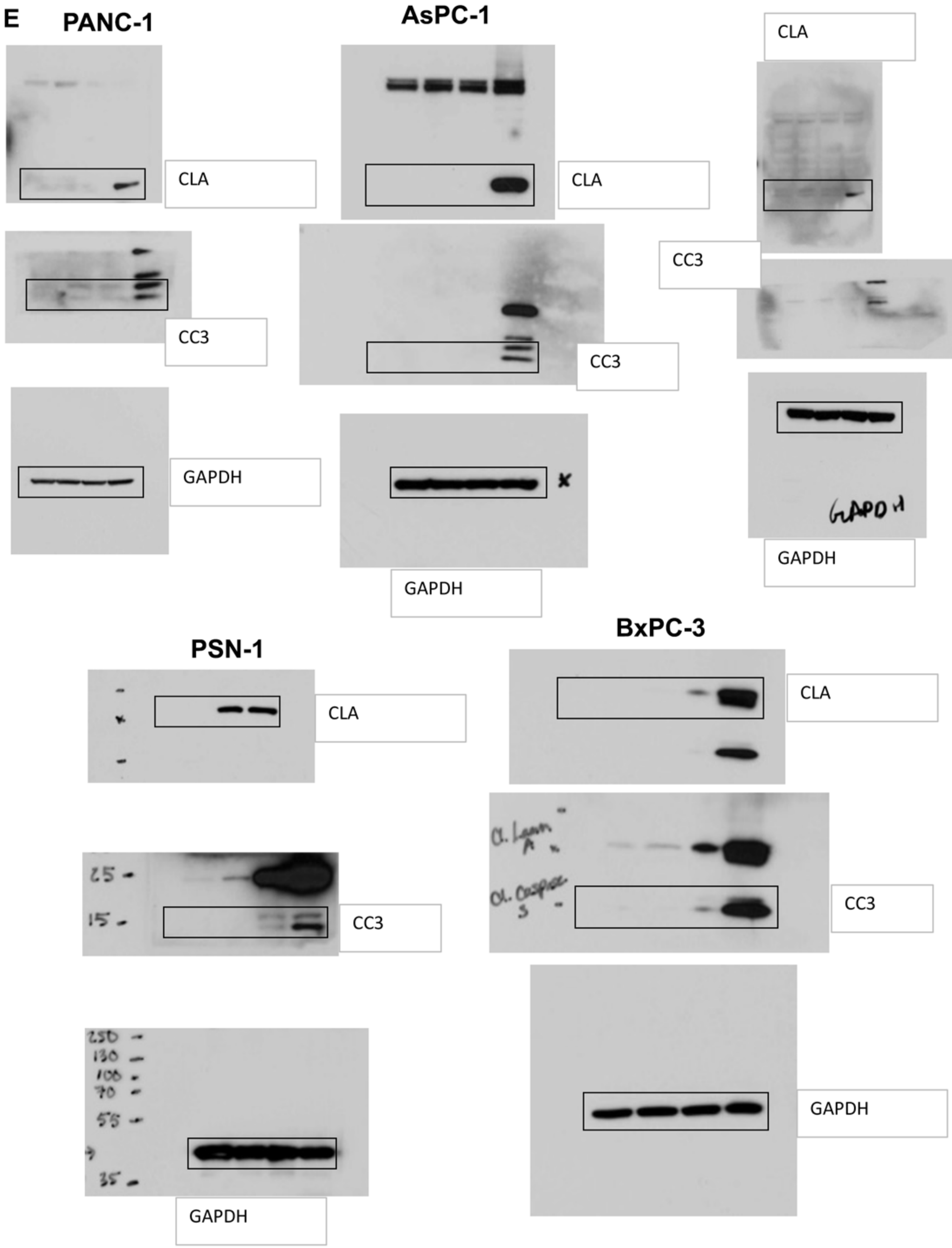

Supplemental Figure 4

F

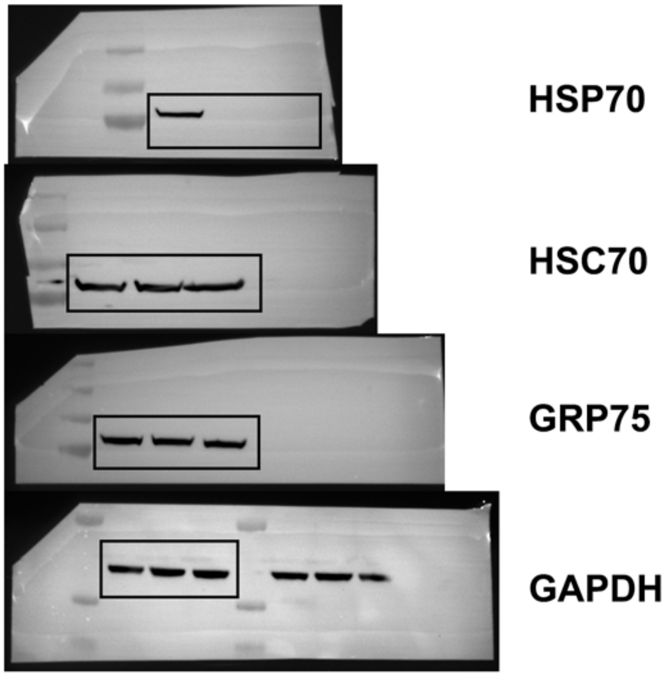

G

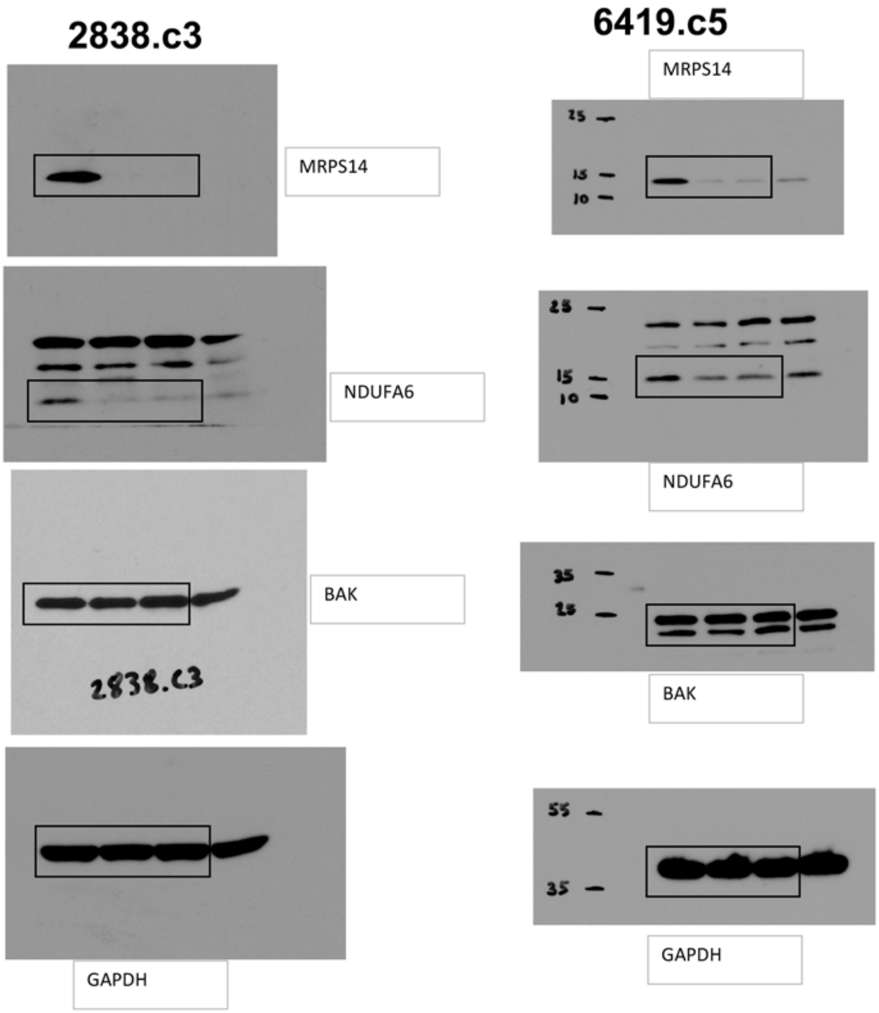

Supplement: Supplementary file 2 — Uncropped Western blots [file 41418_2024_1310_MOESM2_ESM.pdf]
